# Effectiveness of a Worksite Social & Physical Environment Intervention on Need for Recovery, Physical Activity and Relaxation; Results of a Randomized Controlled Trial

**DOI:** 10.1371/journal.pone.0114860

**Published:** 2014-12-26

**Authors:** Jennifer K. Coffeng, Cécile R. L. Boot, Saskia F. A. Duijts, Jos W. R. Twisk, Willem van Mechelen, Ingrid J. M. Hendriksen

**Affiliations:** 1 Department of Public and Occupational Health, EMGO+ Institute for Health and Care Research, VU University Medical Center, Amsterdam, The Netherlands; 2 Body@Work TNO- VU University Medical Center, Research Center Physical Activity, Work and Health, Amsterdam, The Netherlands; 3 Department of Epidemiology and Biostatistics, VU University Medical Center, Amsterdam, The Netherlands; 4 Department of Health Sciences, VU University, Amsterdam, The Netherlands; 5 TNO (Expert Center Life Style), Leiden, The Netherlands; Scottish Collaboration for Public Health Research and Policy (SCPHRP), United Kingdom

## Abstract

**Objective:**

To investigate the effectiveness of a worksite social and physical environment intervention on need for recovery (i.e., early symptoms of work-related mental and physical fatigue), physical activity and relaxation. Also, the effectiveness of the separate interventions was investigated.

**Methods:**

In this 2×2 factorial design study, 412 office employees from a financial service provider participated. Participants were allocated to the combined social and physical intervention, to the social intervention only, to the physical intervention only or to the control group. The primary outcome measure was need for recovery. Secondary outcomes were work-related stress (i.e., exhaustion, detachment and relaxation), small breaks, physical activity (i.e., stair climbing, active commuting, sport activities, light/moderate/vigorous physical activity) and sedentary behavior. Outcomes were measured by questionnaires at baseline, 6 and 12 months follow-up. Multilevel analyses were performed to investigate the effects of the three interventions.

**Results:**

In all intervention groups, a non-significant reduction was found in need for recovery. In the combined intervention (n = 92), exhaustion and vigorous physical activities decreased significantly, and small breaks at work and active commuting increased significantly compared to the control group. The social intervention (n = 118) showed a significant reduction in exhaustion, sedentary behavior at work and a significant increase in small breaks at work and leisure activities. In the physical intervention (n = 96), stair climbing at work and active commuting significantly increased, and sedentary behavior at work decreased significantly compared to the control group.

**Conclusion:**

None of the interventions was effective in improving the need for recovery. It is recommended to implement the social and physical intervention among a population with higher baseline values of need for recovery. Furthermore, the intervention itself could be improved by increasing the intensity of the intervention (for example weekly GMI-sessions), providing physical activity opportunities and exercise schemes, and by more drastic environment interventions (restructuring entire department floor).

**Trial Registration:**

Nederlands Trial Register NTR2553

## Background

Over 22% of European Union employees suffer from stress at work on a daily basis and the annual economic costs of work-related stress in Europe was estimated at 20 billion Euro [1]. The degree to which employees are able to recover from fatigues and stress at work effects their physical and mental health [2].

Results of the Netherlands working condition survey [3], based on a representative sample of the Dutch workforce, revealed that there has been a slight increase in the need for recovery over the years. Recovery after work is a natural consequence of expended effort. However, it becomes problematic when there is not enough recovery offered between two periods of work effort. The effort recovery model developed by Meijman & Mulder (1998) describes the role of recovery [4]. This model depicts job demands and the associated effort expenditure. If job demands continue to strain the individual and no recovery is allowed to occur, excessive load reactions will accumulate, resulting in physical and mental impairment. Recovery occurs when no further effort is needed and load reactions are reduced, so one can return to pre-stressor levels of functioning in which homeostasis of physiological and psychological systems is attained. In line with these findings, the concept need of recovery was introduced. Need for recovery is an early indicator for mentally and physically work-induced fatigue and reflects the need to recuperate and unwind after work [5]. A previous study argued that fatigue can be placed on a continuum from mild to severe fatigue [6]. Conceptually, need for recovery is likely to be located at the beginning of the continuum and is an early precursor for severe, long-term fatigue. Recovery is thus needed to recover from the short-term workload effects of a day at work. A high need for recovery has unfavourable consequences for the individual worker, such as a high blood pressure, sleeping problems and fatigue [7]–[10]. It also predicts future sickness absence lasting fourteen or more working days, which may result in a financial burden for companies due to increased absenteeism [11].

In an effort to reduce the need for recovery, evidence has been found that physical activity is valuable in unwinding from work [12]–[15]. In particular, engaging in physical activity results in lower work stress [16]. When more time is spent on physical activity after work, the feeling of being recovered is heightened [12]. Relaxation is another strategy that seems to be important for recovery. It has been shown that relaxation activities are related to increasing one's feeling of recovery [13], [17]–[20]. A way to achieve relaxation is to disengage from work, which reverses the negative consequences of straining job demands and returns the employee to pre-stressor levels. It was shown that low levels of relaxation are associated with weaker health, emotional exhaustion, a high need for recovery and sleeping problems [17]. In view of this, it is important that interventions will be developed that involve physical activity and relaxation to improve the need for recovery among office employees.

To date, few studies investigated the effectiveness of a WHP program on improving the need for recovery. A study on the effectiveness of a worksite vitality program, consisting of visits of a personal vitality coach and weekly yoga sessions, showed that the intervention group lowered their need for recovery compared to the control group [21] at 6 months follow-up, but this was not sustained in the long-term.

Previous research has shown that, besides changing individual health behavior, both the social and physical environment are important in improving health and well-being [22]–[28], so WHP programs should adopt a socio-ecological model when developing and evaluating interventions. Socio-ecological models focus on making changes to the individual (intrapersonal), social (interpersonal), physical and/or organizational environment. A combined intervention is likely to demonstrate effects over extended periods of time [29]. In the current study, a socio-ecological framed WHP program focussing on a combined social and physical environment intervention was applied, aiming at physical activity and relaxation to improve the need for recovery in office employees. Based on elements of the Intervention Mapping protocol, the intervention program was developed in close cooperation with the employees of a financial service provider with mainly desk jobs. Resulting from a needs assessment (i.e., questionnaire on physical activity, relaxation and need for recovery, individual interviews and focus group interviews with the target population), key determinants of physical activity and relaxation were chosen and, methods and strategies were selected to affect these determinants. This resulted in a social environment intervention consisting of Group Motivational Interviewing (GMI), conducted by teamleaders, and a physical environment intervention consisting of environment modifications (e.g., table tennis and sitting balls).

The objective of this study was to investigate the effectiveness of the combined social and physical environment intervention at 6 and 12 months follow-up compared to a control intervention, as well as the effectiveness of the social environment intervention and the physical environment intervention separately, on need for recovery (i.e., early symptoms of work-related mental and physical fatigue), work-related stress (i.e., exhaustion, detachment and relaxation), small breaks at work, physical activity (i.e., stair climbing, active commuting, leisure activities, sport activities, light/moderate/vigorous physical activity) and sedentary behavior at work. It was hypothesized that the combined intervention would be more effective than the separate interventions compared to the control group.

## Methods

### Study population and study design

The protocol for this trial and supporting CONSORT checklist are available as S1 Checklist and S1 Protocol. Data were used from the Be Active & Relax “Vitality in Practice” (VIP) project (30) conducted at a financial service provider. In September 2011, 1.182 office employees of a financial service provider received an invitation for the Be Active & Relax “Vitality in Practice” (VIP) project. A total of 412 office employees (35% response rate) from 19 departments provided the informed consent, completed the baseline questionnaire and were included in the Be Active & Relax project. All respondents met the inclusion criterion of not being on sick leave for more than four weeks. The participants received follow-up questionnaires at 6 months and 12 months.

The effectiveness of the interventions was investigated in a trial using a 2×2 factorial design. The two factors were the social environment intervention and the physical environment intervention, of which the social environment intervention was randomized at department level and the physical environment intervention was stratified on department level, i.e., one stratum with environment modifications and the other stratum without environment modifications. This resulted in four research groups: (1) combined social and physical environment intervention group; (2) social environment intervention group only; (3) physical environment intervention group only; (4) no intervention (control group). Blinding of the participants and intervention providers for the social environment intervention was impossible, although none of them had received information about our design involving three intervention groups. This study was approved by the Medical Ethics Committee of the VU University Medical Center, Amsterdam, the Netherlands. More details on the study design, methods including all outcome measures and interventions of the Be Active & Relax project have been published elsewhere [30].

### Social and physical environment interventions

#### Social environment intervention

The social environment intervention consisted of Group Motivational Interviewing (GMI) derived from Motivational Interviewing (MI). Motivational interviewing (MI) is a counseling style that stimulates behavioral change by focusing on exploring and resolving ambivalence [31]. It was decided to adjust individual MI to group MI, defined as Group Motivational Interviewing (GMI) [32]–[37]. A group setting has several benefits, e.g., sharing experiences, providing feedback and giving support. GMI helps to create an autonomous supportive environment, in which engaging in daily physical activity and relaxation can be encouraged. This feeling of social support is important to improve one's need for recovery. Previous study by Sluiter 2001 showed that participants with less favorable social relationships at work reported more need for recovery [38].

GMI was delivered by the teamleaders after receiving a two-day training, which was given by a GMI-professional. The trained teamleaders conducted four GMI-sessions of 90 minutes each with employees from their own team, within a period of 3.5 months (i.e., the first 3 sessions were conducted once every three weeks, the last session was after 2 months). The main aim of the sessions was to stimulate physical activity and relaxation, for example during GMI-session two, employees were asked to fill in a worksheet stating their goals and subsequent rewards for improving physical activity and relaxation. The GMI-sessions were supported by a web-based social media platform.

#### Physical environment intervention

Vitality in Practice (VIP) zones were created: (1) the VIP Coffee Corner Zone – the coffee corner was modified by adding a bar with bar chairs, a large plant and a giant wall poster (a poster visualizing a relaxing environment, e.g., wood, water and mountains); (2) the VIP Open Office Zone – the office was modified by introducing exercise balls and curtains to divide desks in order to reduce background noise; (3) the VIP Meeting Zone – conference rooms were modified by placing a standing table (a table that allows you to stand while working) and a giant wall poster (as before); and (4) the VIP Hall Zone - table tennis tables were placed and lounge chairs were introduced in the hall for informal meetings. In addition, footsteps were placed on the floor in the entrance hall to promote stair walking. The physical environment intervention was left in place for the whole 12 months.

**Figure 1:**
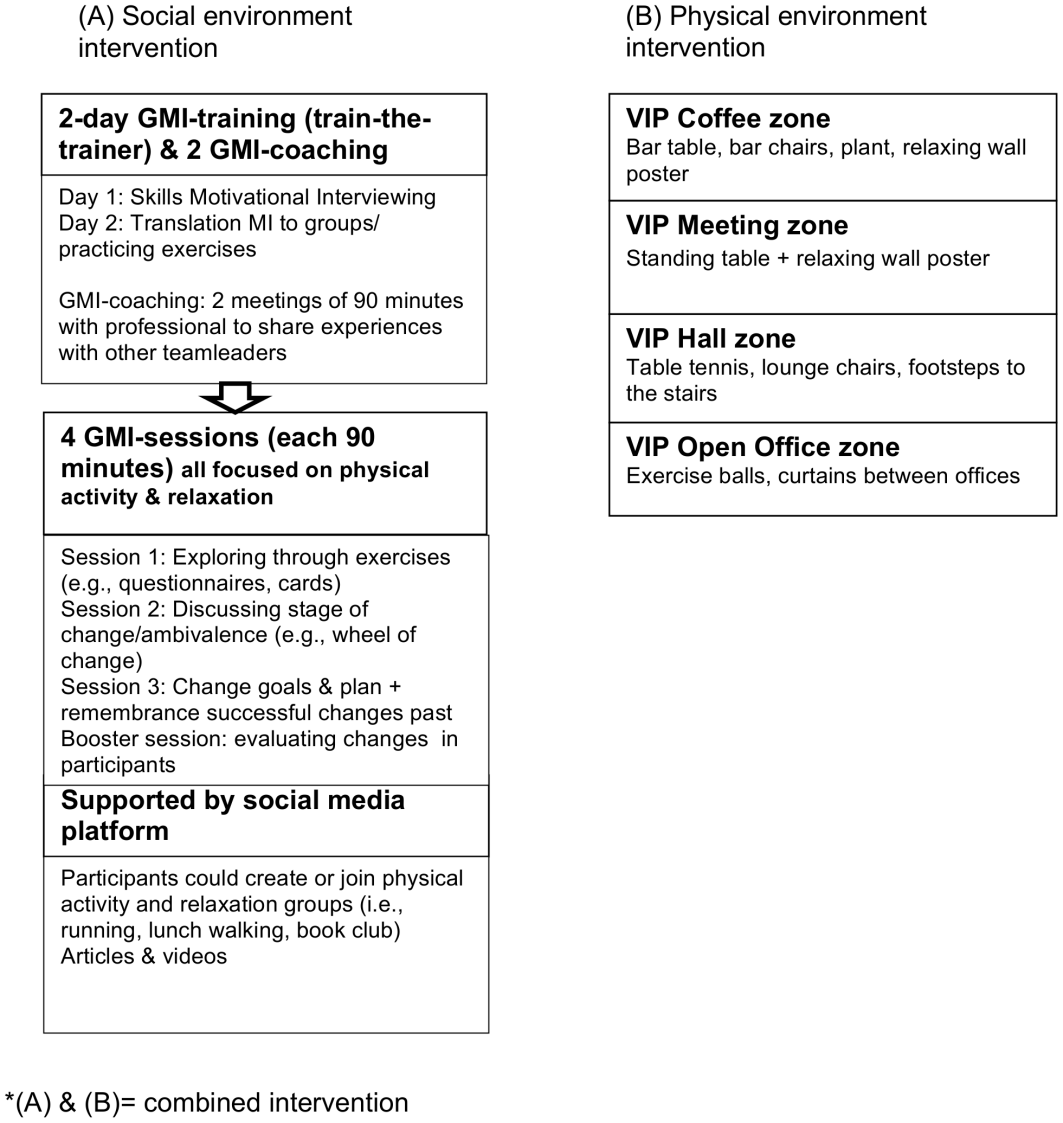
Details intervention. *(A) & (B)  =  combined intervention.

Fig. 1 depicts in more detail the social and physical interventions.

### Outcome measures

*Need for recovery* was assessed using the Need for Recovery after Work scale [39]. This scale consists of eleven dichotomous items (yes/no), representing short-term effects of a day at work, with questions like “I find it hard to relax at the end of a working day” and “When I get home, people should leave me alone for some time”. The need for recovery score is a percentage score (0 to 100) of positive answers of those participants providing data for at least 8 of the 11 items. The need for recovery has shown good internal consistency (α = 0.87) and validity [39]. Validity was studied by analysing the associations of need for recovery with psychosocial risk factors (e.g., emotional load and physical exertion) [39].

#### Sample size

The sample size was calculated based on the number of cases needed to indentify an effect on need for recovery, measured by the Need for Recovery after Work scale of the Dutch VBBA questionnaire [39]. Previous studies of de Croon et al., [40] and Kuijer et al., [41] showed an effect size of 12 (i.e., within a range from 0–100). Average norm score for need for recovery is 38.1 (the anticipated variability; SD = 29.9) on a scale from 0–100 [2]. This anticipated variability indicates that the data are spread out over a quite large range of values. Because of randomisation at department level, a certain loss of efficiency relative to individual randomisation must be considered. For this an intraclass correlation coefficient (ICC) of 0.025 was assumed, based on previous studies showing that worksite level ICC's for health-related outcomes are generally small [42]–[44]. The effect size of 12 can be detected by four groups of 101 participants, taking into account a loss to follow-up of 25%, a power of 80% and a two-tailed significance level of 5%.

#### Work-related stress

*Exhaustion* was measured by the OLdenburg Burnout Inventory (OLBI) consisting of eight items (e.g., “I can usually handle the amount of work well”) on a 4-point scale ranging from “totally agree” to “don't agree” [45]. The OLBI has shown good internal consistency (α = 0.85) and reasonable validity in different occupational groups (i.e., health care workers experienced higher levels of exhaustion than white collar workers) [46].

*Detachment and relaxation* after work were assessed with the Recovery Experience Questionnaire, which was developed by Sonnentag et al. [17]. The validation study of the Recovery Experience Questionnaire [17] resulted in four items measuring detachment (e.g., “I don't think about work at all”) and four items measuring relaxation (e.g., “I use the time to relax”). In the present study, we were also interested in measuring detachment and relaxation during work. For this purpose, the scale was adapted to a within workday context, starting each item with “During a break at work…”, instead of “During time after work…” as written in the original questionnaire of Sonnentag et al. [17]. Each item was assessed on a 7-point scale, ranging from “never” to “always”. The internal consistency and construct validity of these subscales were investigated in a validation study, which was performed alongside the present trial [47]. Internal consistency of the ‘at work’ and ‘after work’ detachment and relaxation scales were considered to be good (Cronbach's alpha ranged from 0.87–0.94). Construct validity was assessed by analysing the associations with need for recovery, exhaustion and work engagement, and was considered to be moderate.

*Small breaks at work* were measured with a newly developed question. Participants were asked how often they engaged in small breaks during a usual workday, using the following question: “Besides your lunch break, how many small breaks (minimum 5 minutes) do you have during an usual workday?”.

#### Physical activity

*Stair climbing at work* was also assessed by a newly developed question. Participants were asked how many times they took the stairs during an usual workday, using the question: “How often do you take the stairs at work during an usual workday?”

Active commuting, leisure activities, sports and total minutes per week in light, moderate and vigorous physical activities was assessed by the Short QUestionnaire to ASsess Health enhancing physical activity (SQUASH) [48]. A previous study [48] reported an overall reproducibility of 0.58 (95%CI, 0.36–0.74), which is comparable to other physical activity questionnaires [49]. Also, reasonable validity of the SQUASH has been demonstrated against accelerometry [48]. The SQUASH questionnaire measures habitual physical activity levels referring to a normal week in the past months in physical activity domains; active commuting (walking and cycling to and from work), physical activity at work, and leisure activities (walking, cycling, gardening, chores and sports). For each domain, employees were asked to report the frequency (times per week), duration of activities (in minutes), and self-reported intensity (light, moderate or vigorous). The leisure domain included information on sports, of which employees could report upon a maximum of four. Physical activity was expressed in minutes per week and total activity scores were calculated by multiplying the minutes per week by the actual MET score (metabolic equivalent, which is the ratio of work metabolic rate to a standard resting metabolic rate of 1.0) of the specific activity (MET/min/week). Information was obtained on light (range <4 MET), moderate (range 4–6.5 MET), and vigorous (>6.5 MET) intensity activities [50].

*Sedentary behavior at work* refers to those activities at work that require a very low energy expenditure (≤1.5 MET) while sitting or reclining [51]. To assess sedentary time at work, participants were asked to estimate the total amount of minutes spend at work on computer use, meetings and other sedentary activities (i.e., making phone calls, reading) during an usual working day. This questionnaire has not been tested for validity yet.

#### Potential confounders

Age, gender, marital status (relationship or single), level of education (low, middle, high education), ethnicity (native or non-native Dutch), and work hours per week were investigated as covariates and potential confounders. Additionally, job demands and supervisor support were assessed on a 4-point scale from “totally agree” to “don't agree” and were derived from the validated Dutch version of the Job Content Questionnaire (JCQ) [52]. General health was measured by one item: “In general, how would you rate your health?” on a 5-point scale, (1 = poor to 5 = excellent) from the Dutch validated version of the Rand-36 [53]. The total scale has shown reasonable validity and satisfactory reliability (α = 0.83).

### Statistical analysis

#### Multilevel regression analyses

To evaluate the intervention effects, we performed a linear mixed model analysis (MlwiN version 2.27) for each outcome measure. Longitudinal data collection often has to deal with missing data due to drop-out of participants. As long as missing at random (MAR) is assumed, mixed model analysis is permitted for incomplete data [54]. We used a linear mixed model analysis with four levels; repeated measures (6 and 12 months) were clustered within employees (n = 365), employees were clustered within team leaders (n = 49) and team leaders were clustered within departments (n = 19). For all levels, only a random intercept was considered. The likelihood ratio test was used to decide whether a random intercept on a particular level should be included in the model.

In the linear mixed models, we compared the four intervention conditions with each other by adding three dummy variables to the model. The control condition was used as the reference group, so that the regression coefficients for the three dummy variables represent the differences between the combined intervention and the control group, between the social intervention and the control group and between the physical intervention and the control group. As such, the regression coefficient of the combined intervention reflects the interaction between the social and the physical intervention directly. For all outcome variables, the following analyses were performed: a crude analysis in which an adjustment was made only for the baseline value of the particular outcome and an adjusted analysis in which an additional adjustment was made for age, gender, education, marital status, general health, job demands and supervisor support. The potential confounders were chosen based on the literature and the expected association with the outcome. In the first set of analyses, overall intervention effects were estimated, while in the second set of analyses, the effects of the interventions at the different time-points were estimated. This was done by adding time (a dichotomous variable differentiating between 6 and 12 months) and the interaction between time and the three dummy variables (representing the three intervention conditions) to the models. Data were analyzed according to the intention-to-treat principle; all participants were analyzed according to the condition, despite the fact whether, and to what extent, they were compliant to the particular intervention and the follow-up measurements. P-values of <0.05 were considered to be significant. The measure of the intervention effect was expressed by betas (B) with 95% confidence intervals.

## Results

### Participants

**Figure 2:**
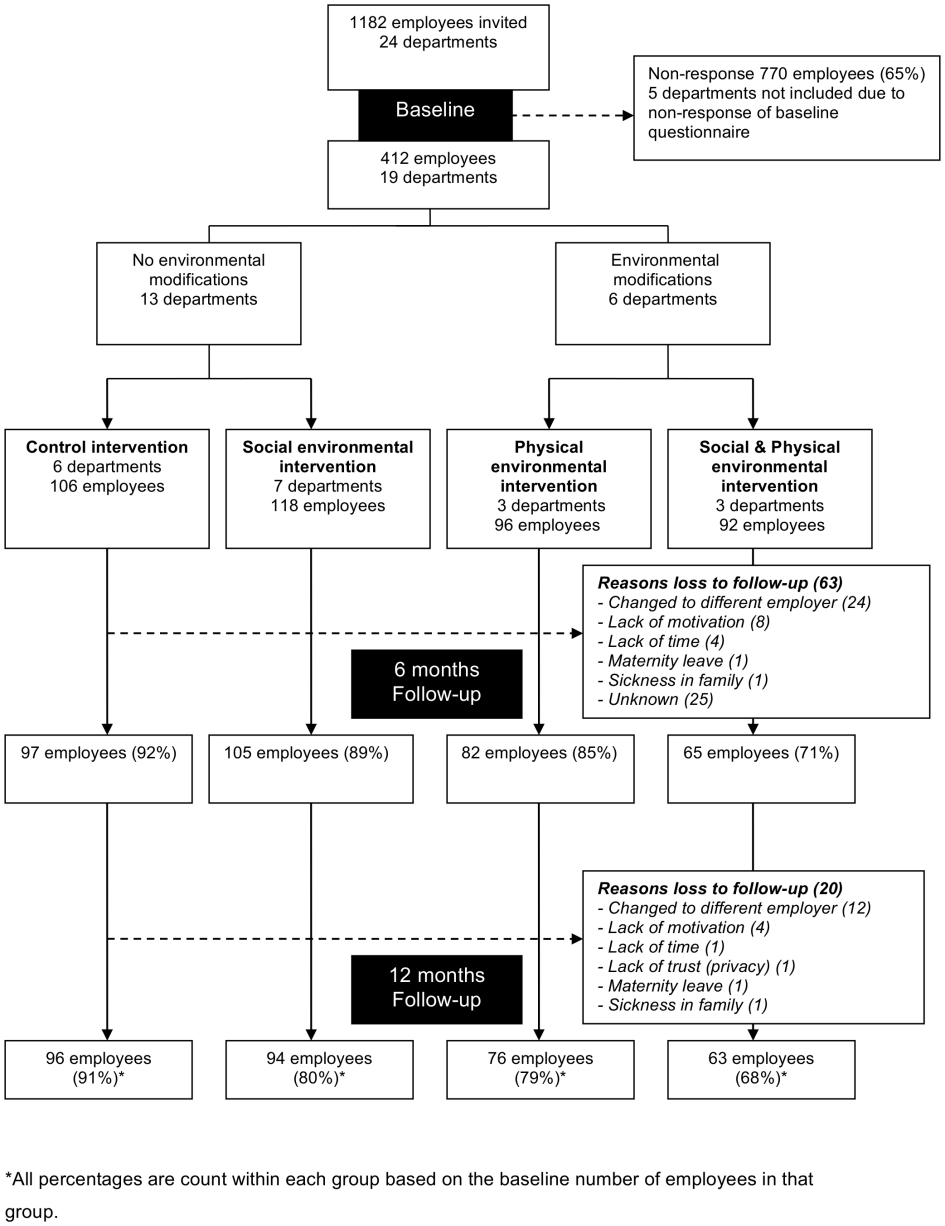
Flow diagram enrolment of participants. *All percentages are count within each group based on the baseline number of employees in that group.

Fig. 2 presents the flow diagram of the study in which 19 departments of the financial service provider participated. Enrolment of the 412 participants took place between September and December 2011. The randomisation procedure allocated 92 employees (from three departments) to the combined intervention, 118 employees (from seven departments) to the social intervention, 96 employees (from three departments) to the physical intervention and 106 employees (from six departments) to the control group. Since randomisation was at department level, the groups were not equal in size. Complete follow-up data was obtained for the primary outcome measure (need for recovery), from 329 participants (80%). For 25 participants, of whom the functions were highly variable, the reasons for loss-to-follow up were unknown. The largest known reason for loss-to-follow up was changing to a different employer (n = 24).

The baseline characteristics of the office employees in the intervention and control groups are given in Table 1. No differences regarding age, gender, education, marital status, ethnicity, working hours, general health, job demands, supervisor support were observed between the intervention groups and control group. However, among the participants, males were slightly overrepresented (60%) and the majority was highly educated (57%). The top 3 functions were; insurance acceptants (13.3%), team leaders (11.4%) and administrative employees (9.4%). Further, 75% of the participants stated to have high job demands and 75.7% reported to have social support at work.

**Table 1:** Baseline characteristics of the study population.

		Social & Physical environment intervention	Social environment intervention	Physical environment intervention	No intervention (control)
Baseline characteristics		n = 92	n = 118	n = 96	n = 106
Nr of departments		3	7	3	6
Male [n (%)]		51 (55.4)	73 (61.9)	60 (62.5)	65 (61.3)
Age (years)^a^		38.0 (10.5)	43.6 (10.3)	42.2 (10.5)	40.7 (9.2)
Having a partner [n (%)]		74 (80.4)	91 (77.1)	82 (85.4)	85 (80.2)
Dutch nationality [n (%)]		82 (89.1)	106 (89.8)	87 (90.6)	95 (89.6)
Education level [n (%)]	Low	17 (18.7)	39 (33.1)	16 (16.7)	21 (19.8)
Intermediate		19 (20.9)	23 (19.5)	20 (20.8)	24 (22.6)
High		55 (60.4)	56 (47.5)	60 (62.5)	61 (57.5)
Working hours per week^a^		35.1 (6.1)	36.9 (4.1)	35.7 (5.6)	36.2 (5.3)
General health (range:1–5)^a^		3.8 (0.9)	3.8 (0.7)	3.8 (0.7)	3.8 (0.7)
Job demands (range:1–5)^a^		2.6 (0.3)	2.7 (0.2)	2.6 (0.3)	2.7 (0.3)
Supervisor support (range:1–5)^a^		2.8 (0.5)	2.9 (0.5)	2.9 (0.4)	2.9 (0.5)

a Mean (Standard Deviation); n =  number of employees.

The means of need for recovery, exhaustion, detachment, relaxation, small breaks, stair climbing, physical activity and sedentary behavior for work at baseline and follow-up measurements are presented in Table 2.

**Table 2:** Means and standard deviations of all outcome measures for the intervention groups versus the control group at baseline, 6 months and 12 months.

	Social & Physical environment intervention			Social environment intervention			Physical environment intervention			No intervention (control)		
	n	M	SD	n	M	SD	n	M	SD	n	M	SD
**Need for recovery (range:0–100)**												
Baseline	92	33.3	29.9	118	31.8	28.7	96	33.7	31.3	106	30.4	27.7
6 months	65	29.4	27.2	105	29.7	28.3	82	30.3	29.1	97	31.9	30.1
12 months	63	24.0	24.5	94	28.1	30.3	76	29.3	29.8	96	28.8	29.1
***Work-related stress*** ** Exhaustion (range:1–4)**												
Baseline	92	2.3	0.5	118	2.1	0.5	96	2.1	0.5	106	2.1	0.5
6 months	57	2.2	0.4	95	2.3	0.4	68	2.2	0.5	83	2.3	0.5
12 months	54	2.2	0.4	78	2.1	0.4	65	2.1	0.5	85	2.2	0.5
**Detachment at work (range:1–7)**												
Baseline	92	3.4	1.4	118	3.5	1.5	95	3.6	1.3	106	3.4	1.4
6 months	53	2.3	1.2	84	2.6	1.5	56	2.7	1.5	79	2.5	1.5
12 months	49	2.4	1.5	74	2.6	1.5	56	2.5	1.3	75	2.6	1.5
**Detachment after work (range:1–7)**												
Baseline	92	4.7	1.4	118	4.8	1.3	96	4.8	1.4	106	4.8	1.3
6 months	53	3.8	1.2	83	4.0	1.1	56	3.9	1.3	78	3.8	1.4
12 months	49	3.7	1.3	73	4.0	1.2	55	4.0	1.1	75	3.9	1.4
**Relaxation at work (range:1–7)**												
Baseline	92	3.5	1.3	118	3.5	1.3	95	3.5	1.2	106	3.9	1.4
6 months	53	2.5	1.2	84	2.7	1.4	56	2.9	1.5	79	2.8	1.4
12 months	49	2.5	1.1	74	2.8	1.5	56	2.6	1.2	75	2.9	1.5
**Relaxation after work (range:1–7)**												
Baseline	92	5.0	1.1	117	5.3	1.0	96	5.2	1.1	106	5.3	1.1
6 months	53	4.0	1.0	83	4.2	1.2	56	4.2	1.1	78	4.1	1.3
12 months	49	4.1	1.0	73	4.3	1.1	55	4.3	1.1	75	4.2	1.3
**Small breaks at work (range:0–30)**												
Baseline	88	1.5	1.5	116	1.4	1.6	96	1.6	1.6	106	1.6	1.7
6 months	53	2.5	2.1	84	2.5	2.6	57	1.6	1.4	79	1.8	1.9
12 months	48	2.7	2.6	74	2.1	3.6	56	1.5	1.5	75	2.0	2.7
***Physical activity*** ** Stair climbing at work (range:0–20)**												
Baseline	90	4.1	2.8	108	1.9	1.9	96	3.4	2.4	99	2.5	2.0
6 months	53	4.2	2.9	88	1.8	1.9	59	4.6	3.0	80	2.3	2.2
12 months	49	4.0	2.8	73	1.7	1.8	57	4.0	2.9	78	2.0	1.7
**Active commuting (minutes per week)**												
Baseline	92	72.4	95.9	118	91.7	130.0	96	82.5	121.0	106	97.5	94.6
6 months	91	373.6	893.8	90	261.2	628.4	86	141.6	281.9	82	263.4	564.5
12 months	63	293.4	788.5	94	145.7	222.2	76	434.1	1235.3	96	152.0	161.8
**Leisure activities (minutes per week)**												
Baseline	92	343.8	279.1	118	336.5	273.3	96	363.7	297.9	106	422.0	292.8
6 months	91	224.7	314.5	90	304.0	727.9	86	208.9	236.2	82	276.3	285.3
12 months	63	389.8	360.8	94	523.9	898.2	76	449.4	531.6	96	421.6	382.9
**Sport activities (minutes per week)**												
Baseline	92	173.5	221.8	118	149.8	193.9	96	167.8	180.9	106	188.8	190.4
6 months	65	144.5	230.3	105	147.4	211.6	82	145.5	215.1	97	142.3	220.2
12 months	63	122.6	146.7	94	203.9	501.0	76	145.6	202.7	96	167.5	244.5
**Light physical activity (minutes per week)**												
Baseline	92	1774.7	1229.0	118	1760.5	1497.4	96	1683.1	1403.4	106	2011.1	1276.8
6 months	91	1788.9	1697.4	90	1448.2	1549.6	86	1481.3	1320.3	82	1539.0	1399.2
12 months	63	1672.8	1551.7	94	1447.0	1575.2	76	1409.4	1659.7	96	1864.0	1657.6
**Moderate physical activity (minutes per week)**												
Baseline	92	239.5	186.2	118	252.5	243.3	96	263.0	251.4	106	368.2	298.8
6 months	91	263.7	502.0	90	327.5	536.6	86	180.8	208.8	82	240.7	269.4
12 months	63	371.2	687.3	94	357.6	740.9	76	349.8	697.3	96	332.0	397.0
**Vigorous physical activity (minutes per week)**												
Baseline	92	84.9	205.5	118	87.5	172.6	96	75.2	124.3	106	85.5	128.5
6 months	91	7.3	42.4	90	28.7	97.5	86	48.1	285.8	82	52.3	189.4
12 months	63	75.1	122.5	94	121.6	432.0	76	100.3	174.9	96	94.2	198.9
**Sedentary behavior at work (minutes per day)**												
Baseline	92	477.3	166.4	118	472.2	148.8	96	500.8	170.4	106	471.3	149.3
6 months	63	380.6	221.6	100	428.5	215.9	76	359.7	262.1	93	414.6	209.2
12 months	63	378.6	221.8	94	365.8	239.2	76	367.2	249.6	96	403.8	245.2

n =  number of employees; M =  Mean; SD =  Standard Deviation.

### Intervention effects on need for recovery

The results on the primary outcome measure of the study, need for recovery, are presented in Table 3. All the interventions showed a reduction in need for recovery (β: range −2.4 to −6.8 at 12 months on a scale of 0–100). The largest reduction was found in the combined intervention at 12 months (β: −6.8, 95%CI: −14.0; −0.4). However, neither the overall intervention effects nor the effects at any time point were significant for need for recovery.

**Table 3:** Crude and adjusted overall effects, at 6 months and at 12 months, of need for recovery between the intervention groups and the control group.

	Social & Physical environment intervention			Social environment intervention			Physical environment intervention		
Need for recovery (0–100)	B	95% CI	p-value	B	95% CI	p-value	B	95% CI	p-value
Overall crude	−4.6	−11.3–2.0	0.17	−1.9	−7.9–4.1	0.53	−3.4	−9.8–3.0	0.30
Overall adjusted	−4.8	−11.4–1.9	0.16	−1.8	−7.8–4.2	0.56	−3.5	−9.8–2.9	0.29
6 months crude	−2.6	−9.7–4.5	0.48	−1.5	−7.8–4.8	0.65	−2.9	−9.6–3.8	0.40
6 months adjusted	−2.9	−10.0–4.3	0.43	−1.4	−7.7–4.9	0.67	−2.8	−9.6–3.9	0.41
12 months crude	−6.8	−14.0–0.4	0.06	−2.4	− 8.8–3.9	0.45	−3.9	−10.8–2.9	0.26
12 months adjusted	−6.8	−14.0–0.4	0.07	−2.3	−8.7–4.2	0.49	−4.2	−11.0–2.7	0.23

Adjusted for confounders age, gender, education, marital status, general health, job demands, supervisor support, and corresponding baseline measure of the outcome variable. Significant effects are in bold. A negative Bèta (B) means a lower need for recovery in the intervention group compared to the control group. [B =  Bèta, CI =  Confidence Interval, p-value is significant <0.05].

For the other outcomes, the overall effects of the interventions are shown in Table 4. When found significant at the separate time points (i.e., 6 and 12 months), these effects are presented in Table 5.

**Table 4:** Crude and adjusted overall effects in all secondary outcome measures between the intervention groups and the control group over a 12 months follow-up period.

	Social & Physical environment intervention			Social environment intervention			Physical environment intervention		
	B	95% CI	p-value	B	95% CI	p-value	B	95% CI	p-value
***Work-related stress*** ** Exhaustion (1–4)** Crude	**−0.2**	**−0.3–−0.1**	**<0.01**	−0.1	−0.1–0.0	0.13	−0.1	−0.2–0.0	0.16
Adjusted	**−0.2**	**−0.3–−0.1**	**<0.01**	−0.1	−0.1–0.0	0.13	−0.1	−0.2–0.0	0.23
**Detachment at work (1–7)** Crude	−0.1	−0.5–0.3	0.58	0.1	−0.3–0.4	0.76	0.0	−0.3–0.4	0.90
Adjusted	−0.2	−0.5–0.2	0.41	0.1	−0.3–0.4	0.76	0.1	−0.3–0.4	0.77
**Detachment after work (1–7)** Crude	0.0	−0.3–0.3	0.85	0.2	−0.1–0.4	0.23	0.2	−0.1–0.5	0.29
Adjusted	0.0	−0.3–0.3	0.85	0.1	−0.1–0.4	0.35	0.2	−0.1–0.5	0.16
**Relaxation at work** **(1–7)** Crude	−0.2	−0.6–0.1	0.24	0.0	−0.3–0.3	0.85	0.1	−0.3–0.4	0.68
Adjusted	−0.3	−0.6–0.0	0.09	0.1	−0.3–0.4	0.70	0.1	−0.3–0.4	0.64
**Relaxation after work (1–7)** Crude	0.1	−0.2–0.4	0.52	0.2	−0.1–0.4	0.25	0.2	−0.1–0.5	0.15
Adjusted	0.1	−0.2–0.4	0.55	0.1	−0.1–0.4	0.25	0.2	−0.1–0.5	0.12
**Small breaks at work (0–30)** Crude	**0.8**	**0.2–1.3**	**<0.01**	0.4	−0.1–0.9	0.08	−0.2	−0.7–0.3	0.44
Adjusted	**0.8**	**0.3–1.3**	**<0.01**	0.4	−0.1–0.9	0.08	−0.2	−0.7–0.3	0.47
***Physical activity*** ** Stair climbing at work (0-20)** Crude	0.4	−0.2–0.9	0.19	−0.2	−0.7–0.3	0.40	**1.0**	**0.5–1.5**	**<0.01**
Adjusted	0.5	−0.0–1.1	0.05	−0.1	−0.6–0.4	0.63	**1.0**	**0.5–1.5**	**<0.01**
**Active commuting** A Crude	144.1	−1.5–289.6	0.05	2.01	−136.3–140.3	0.98	81.2	−62.2–224.6	0.27
Adjusted	142.0	−5.5–289.4	0.06	−8.3	−150.0–133.4	0.91	91.9	−53.8–237.5	0.22
**Leisure activities** A Crude	−47.8	−162.4–66.7	0.42	71.0	−38.1–180.0	0.20	−20.8	−133.6–92.0	0.72
Adjusted	−41.5	−155.9–72.8	0.48	33.5	−76.9–144.0	0.55	−28.0	−141.1–85.2	0.63
**Sport activities** A Crude	−18.8	−76.8–39.3	0.53	35.5	−16.2–87.1	0.18	5.9	−48.8–60.6	0.83
Adjusted	−18.9	−77.1–39.3	0.52	30.4	−22.0–82.7	0.25	3.6	−51.2–58.6	0.90
**Light physical activity** A Crude	10.5	−344.8–366.0	0.95	−324.0	−660.7–12.7	0.06	−211.8	−563.8–140.3	0.24
Adjusted	−37.3	−396.8–322.2	0.84	−322.5	−665.5–20.5	0.07	−217.1	−573.6–139.4	0.23
**Moderate physical activity** A Crude	57.7	−5.8–173.5	0.32	87.8	−22.2–197.8	0.12	6.5	−107.4–120.4	0.91
Adjusted	54.8	−58.1–171.8	0.36	63.1	−48.9–175.2	0.27	6.8	−108.1–121.7	0.90
**Vigorous physical activity** A Crude	−42.2	−91.1–6.8	0.09	−1.4	−48.0–45.3	0.51	−0.2	−48.4–48.0	0.99
Adjusted	−38.5	−88.0–11.0	0.13	−11.6	−59.3–36.2	0.64	−4.6	−53.2–44.0	0.86
**Sedentary behavior at work**B Crude	−33.9	−90.6–22.9	0.24	−16.9	−67.4–33.7	0.52	**−54.6**	**−108.6–−0.5**	**0.048**
Adjusted	−33.8	−90.3–22.7	0.24	−29.8	−80.3–20.8	0.29	**−57.9**	**−111.7–−4.2**	**0.03**

A minutes per week, ^B^minutes per day. Adjusted for confounders age, gender, education, marital status, general health, job demands, supervisor support, and corresponding baseline measure of the outcome variable. Significant effects are in bold;. A negative Bèta (B) means less exhaustion, detachment and relaxation at work and after work, small breaks, stair climbing, active commuting, leisure activities, sports, light physical activity, moderate physical activity, vigorous physical activity and sedentary behavior in the intervention group compared to the control group. [B =  Bèta, CI =  Confidence Interval, p-value is significant <0.05].

**Table 5:** Crude and adjusted effects on exhaustion, small breaks, stair climbing, active commuting, leisure activities, sport activities, vigorous physical activity, sedentary behavior at work between the intervention groups and control group found significant at 6 and/or 12 months follow-up.

	Social & Physical environment intervention			Social environment intervention			Physical environment intervention		
	B	95% CI	p-value	B	95% CI	p-value	B	95% CI	p-value
***Work-related stress*** ** Exhaustion (1–4)** 6 months crude	**−0.2**	**−0.0–−0.3**	**<0.01**	−0.0	−0.1–0.1	0.97	−0.0	−0.1–0.1	0.5
6 months adjusted	**−0.2**	**−0.0–−0.3**	**<0.01**	0.0	−0.1–0.1	0.99	−0.0	−0.1–0.1	0.5
12 months crude	**−0.2**	**−0.1–−0.3**	**<0.01**	**−0.1**	**−0.2–−0.0**	**<0.01**	−0.1	−0.2–0.0	0.06
12 months adjusted	**−0.2**	**0.1–1.3**	**<0.01**	**−0.1**	**−0.2–−0.0**	**<0.01**	−0.1	−0.2–0.0	0.06
**Small Breaks at work (0–30)** 6 months crude	**0.7**	**0.1–1.3**	**0.02**	**0.7**	**0.2–1.2**	**<0.01**	−0.1	−0.6–0.5	0.84
6 months adjusted	**0.8**	**0.1–1.4**	**0.02**	**0.7**	**0.2–1.2**	**<0.01**	−0.1	−0.7–0.5	0.74
12 months crude	**0.8**	**0.1–1.4**	**0.01**	0.1	**−**0.4–0.7	0.67	−0.3	−0.9–0.2	0.26
12 months adjusted	**0.8**	**0.2–1.5**	**0.01**	0.1	−0.4–0.6	0.72	−0.3	−0.9–0.3	0.38
***Physical activity*** ** Stair climbing at work (0-20)** 6 months crude	0.4	−0.2–1.0	0.19	−0.1	−0.7–0.4	0.63	**1.3**	**0.7–1.8**	**<0.01**
6 months adjusted	0.6	−0.0–1.2	0.07	−0.0	−0.6–0.5	0.94	**1.3**	**0.7–1.8**	**<0.01**
12 months crude	0.3	−0.3–1.0	0.32	−0.3	−0.9–0.2	0.25	**0.7**	**0.1–1.3**	**0.02**
12 months adjusted	0.5	−0.1–1.1	0.13	−0.2	−0.8–0.3	0.41	**0.8**	**0.2–1.3**	**0.01**
**Active commuting** A 6 months crude	**177.2**	**8.2–346.1**	**0.04**	60.0	−108.5–228.6	0.48	−55.3	−227.2–116.6	0.53
6 months adjusted	**175.1**	**4.3–346.0**	**<0.05**	50.1	−121.3–221.5	0.57	−52.8	−227.3–121.8	0.56
12 months crude	96.9	−95.1–288.8	0.32	−53.5	−219.5–112.6	0.53	**234.1**	**55.4–412.8**	**0.01**
12 months adjusted	98.1	−95.9–292.2	0.32	−66.3	−236.1–103.5	0.45	**252.1**	**71.0–433.3**	**<0.01**
**Leisure activities** A 6 months crude	49.4	−100.4–199.1	0.52	**180.9**	**60.0–310.8**	**<0.01**	106.9	−32.4–246.3	0.13
6 months adjusted	48.2	−101.5–198.0	0.53	**132.9**	**1.4–264.4**	**<0.05**	48.2	−101.5–198.0	0.53
**Sport activities** A 12 months crude	−32.7	−103.5–38.0	0.36	**65.8**	**4.2–127.4**	**0.04**	5.0	−61.1–71.0	0.88
12 months adjusted	−33.7	−104.9–37.6	0.35	59.9	−2.7–122.5	0.06	1.4	−65.2–67.9	0.73
**Vigorous physical activity** A 6 months crude	**−64.9**	**−123.3–−6.5**	**0.03**	−43.4	−101.6–15.1	0.14	−24.3	−84.3–35.9	0.43
6 months adjusted	**−62.6**	**−120.9–−3.9**	**0.03**	−53.6	−111.3–4.1	0.07	−30.5	−89.2–28.2	0.31
**Sedentary behavior at work** B 12 months crude	−36.1	−103.6–31.4	0.29	−50.1	−108.8–8.7	0.10	−53.5	−116.7–9.7	0.10
12 months adjusted	−36.0	−103.3–31.3	0.29	**−66.2**	**−125.4–−7.0**	**0.03**	−61.4	−124.5–1.7	0.06

A minutes per week,

B minutes per day. Adjusted for confounders age, gender, education, marital status, general health, job demands, supervisor support, and corresponding baseline measure of the outcome variable. Significant effects are in bold. A negative Bèta (B) means less exhaustion, small breaks, stair climbing, active commuting, leisure activities, sport activities, vigorous physical activity, sedentary behavior in the intervention group compared to the control group. [B =  Bèta, CI =  Confidence Interval, p-value is significant <0.05].

### Work-related stress outcomes

The interventions did neither result in any significant effects for detachment, nor for relaxation at work and after work. The combined intervention was associated with a significant lower overall level of exhaustion (β: −0.2, 95%CI: −0.03; −0.1), which was persistent at 6 and 12 months.

### Small breaks

Both the combined and the social intervention showed a significant increase in small breaks at work (β: range 0.7 to 0.8 on a scale of 0–30). For the combined intervention this significant effect was persistent at 6 and 12 months, for the social intervention significancy persisted only at 6 months.

### Physical activity outcomes

The interventions did not result in any significant effect for light or moderate intensity physical activity. The combined intervention showed a significant increase in active commuting (β: 175.1, 95%CI: 4.3; −346.0) and a reduction in vigorous physical activity at 6 months (β: −62.6, 95%CI: −120.9; −3.9). The social intervention showed a significant increase in leisure activities at 6 months (β: 132.9, 95%CI: 1.4; 264.4). In the physical intervention, an overall increase in stair climbing at work was found (β: 1.0, 95%CI: 0.5; 1.5), which was persistent at both 6 and 12 months follow-up. Further, a significant increase in active commuting was found at 12 months (β: 252.1, 95%CI: 71.0; 433.3).

### Sedentary time at work

The social intervention showed a significant reduction in sedentary time at 12 months (β: −66.2, 95%CI: −125.4; −7.0). For the physical intervention, an overall reduction in sedentary time at work was found (β: −57.9, 95%CI: −111.7; −4.2), but no effects were found at the two respective follow-up periods.

## Discussion

The results of this study showed that none of the interventions was effective in improving the need for recovery among office workers compared to the control group. Nevertheless, all outcomes changed into the expected, favourable direction. We did find statistically significant, but small, decreases in exhaustion, vigorous physical activity and sedentary behavior at work, and statistically significant increases in small breaks at work, active commuting, stair climbing at work, and leisure activities.

### Need for recovery

Based on the literature, it was hypothesized that our combined social and physical intervention would be effective in improving the need for recovery. Our findings did not confirm this hypothesis, however, we found the largest reduction in the combined intervention (β: −6.8, 95%CI: −14.0; −0.4). Although literature on this topic is scarce, this non-significant result is in line with two recent other studies: one among construction employees (that provided empowerment training and training guided by a physical therapist) [55] which showed an odds ratio of 0.88 at 12 months (an odds ratio (OR) above 1 indicates that employees in the intervention group had on average higher need for recovery compared to the control group). Another study among employees of two Dutch research institutes (mindfulness intervention) showed a non-significant increase at 12 months (β: 1.3, 95%CI: −3.7; 6.3) [56]. Even more scarce are studies involving the effect of changes in the physical environment on need for recovery. Only one study was found [57], where the effects of a so-called innovative office concept (e.g., open-office plan and flexible workplaces) on need for recovery among Dutch office employees was studied. They have found no significant improvements in need for recovery (mean at T0: 22.6 and at T2: 22.3) at 15 months follow-up [57].

The present study directed efforts at a healthy population, instead of a population with mental or physical health problems [58]. A downside of this focus is that it may offer relatively small health benefits and subjects are less motivated [59]. The participants of our study had relatively low baseline values (i.e., favourable) on need for recovery (M = 33.2, SD = 29.3), compared to average norm scores (M = 38.1) [60]. A possible explanation for this finding might be that employees with healthy lifestyles are more likely to participate, because they are more motivated to pursue and maintain their good health [61]. Due to these favourable baseline values, a significant decline in need for recovery as a result of the intervention is difficult to obtain (bottom effects). To have larger effects, efforts should be directed at a selection of participants with high need for recovery.

### Work-related stress

Baseline values for exhaustion were generally low in all intervention groups and in the control group. Nevertheless, in the combined intervention, we did find a small significant reduction, which was present both at 6 months and at 12 months. In the social intervention, a small significant reduction at 12 months was found. The effect was considered not relevant, because it was smaller than 10%. Exhaustion was measured using the OLdenburg Burnout Inventory (OLBI) [45]. Information about norm scores of this scale is unknown. A study among healthcare employees, recruited on the basis of a high level of exhaustion, found that peer-support groups could be helpful in reducing levels of exhaustion, stress and minimizing work-family conflict [62]. This offers a possible explanation for a small reduction in exhaustion in the combined and social intervention, because both team leaders and colleagues had an active role in stimulating and supporting each other. No such a reduction of exhaustion was found in the physical intervention.

With respect to detachment and relaxation, no effects were found. Although mental separation through detachment or relaxation has attracted attention, because it is helpful in improving job performance [63] and well-being [64], it is not very often considered in WHP programs. Understanding the particular activities people execute for detachment and relaxation, and their interrelationships at work and after work, is important for future intervention development. Further, it could be that employees did not feel legitimized to detach or relax during work hours, which could explain that no effects were found on detachment and relaxation. Also, alongside the present trial, a study was executed assessing the measurement properties of the detachment and relaxation scale. The results showed that the scale was internally consistent, reliable and had moderate construct validity [47]. The lack of effect might be explained by the fact that unsatisfactory responsiveness was demonstrated [47]. Therefore, conclusions on detachment and relaxation based on our present results must be drawn with care.

### Small breaks

We did find small significant improvements (β: range 0.7 to 0.8 on a scale of 0–30) in the frequency of small breaks at work in the combined intervention at both time points, and in the social intervention at 6 months. So far, few RCTs have been conducted that investigated the effectiveness of WHP programs on small breaks. In the area of work recovery, one study showed that enjoyable and restful within workday breaks improved the recovery in a group of service employees [65]. Since the short-term benefits of within workday breaks are restorative, refreshing and energizing [66], our small improvement in small breaks (less than 10%) are a step up for future research.

### Physical activity

A higher amount of minutes in active commuting and a reduction in vigorous physical activity at 6 months was found in the combined intervention. In the social intervention, the amount of time spent in leisure activities at 6 months was largely increased. The physical intervention showed an increase in stair climbing and active commuting at 6 and 12 months. We did find an unexpected, relatively small, significant reduction in vigorous physical activity at 6 months in the combined intervention. Since we did not focus on vigorous physical activity in particular, it is possible that participants focussed on light to moderate physical activity, therewith reducing their focus on vigorous physical activity.

### Sedentary time at work

Furthermore, a small significant reduction (β: range −57.9 to −66.2) in minutes of sedentary time was shown in the social and the physical intervention, but not in the combined intervention. Previous studies have shown that high amounts of sedentary time increase the risk of morbidity and mortality, irrespective of whether people engage frequently in moderate to vigorous intensity activities [67], [68]. Although the questionnaire on sedentary behavior during work is used more often in worksite health promotion intervention studies, it has not been validated. This may have resulted in less reliable findings and the results should be interpreted with caution.

### Strengths and limitations

A strength of our study is our study design, in which we applied a 2×2 factorial design in which we could randomise the social environment intervention and stratify the physical environment intervention. This enabled us to simultaneously study the effectiveness of the combined, and the social and the physical environment interventions separately. Another strength is the minimized risk of contamination due to randomisation at department level. Contamination could be of concern when the control group is able to mingle with the intervention group and therefore receive also whole or part of the intervention. Through departmental randomization, cross-over was not expected to be likely. For GMI, no level of contamination was expected as the control group did not have access to GMI. However, it could be that the environment modifications such as table tennis and lounge chairs were also used by the control group.

Moreover, during all analyses, the multilevel procedure was applied. With multilevel analyses, incompleteness of the data is taken into account (i.e., method of maximum likelihood), and imputation of missing data is thus not necessary [69]. The loss-to-follow up at 6 months for the secondary outcome measures (>20%) was considerable, which is a common problem in intervention studies [70]. However, there were no significant differences at baseline between non-responders and responders, and therefore it is unlikely that self-selection of participants has influenced our study results.

Further, the present study did not account for cluster-level confounding. However, because we have added random intercepts for the different levels, we adjusted for confounding for that particular level. Nevertheless, there can be some uncontrolled residual confounding on each level which can lead to biased variance estimates and thus biased study inference [71].

According to the executed process evaluation [72], the reach (percentage of participants that used any of the intervention components at least once) for the social and physical intervention ranged between 45–76% and was considered to be reasonable, compared to other workplace health promotion programs (mostly below 50%). A limitation is that reach was assessed by self-report and no objective measurements were used. To illustrate, we were not continuously present to monitor the reach of the social and physical intervention and therefore we needed to rely on self-report information of the participants. As a result, we do not have an appropriate level of reach for both the social and physical intervention.

Some considerations should be given to the measurement properties of the questionnaires used. One issue concerns recall bias in measuring small breaks or detachment/relaxation, because previous research indicated that strenuous activities are more easily recalled than light activities [73]. Also, previous research showed that self-reported stair use was systematically overestimated [74] and self-reported physical activity levels proved to show less accurate information on actual physical activity levels than objectively measured physical activity using accelerometry [75].

Unfortunately, the present study was underpowered regarding the primary outcome measure, need for recovery. Although our total sample size was sufficient, our smallest intervention group contained 63 instead of the anticipated 76 participants due to a loss-to-follow up at 6 months (29%) and at 12 months (32%). This may have contributed to the fact that we did not find a significant effect on need for recovery in the combined intervention group. However, we do not expect that only a larger sample size would give other results since related studies in ergonomics did not had significant effects either. For example, in a study by Feuerstein et al., [76] on the effectiveness of a single ergonomic approach and of a combined approach offering a job stress management program: comparable to our study the combined approach was not more effective. This was also seen in a study of Bernaards et al., [77] in which the effectiveness of a workstyle intervention and a workstyle intervention combined with physical activity was evaluated. Also, this study did not show that the combined intervention was more effective compared to the workstyle intervention alone. An explanation for these findings and the findings of our study is that a combination of interventions could have resulted in a lack of focus, resulting in smaller behavioural changes. Or antagonism of effects could have played a role.

Further, we made use of a minimal intervention strategy for the GMI-intervention, i.e., a two-day training in GMI, and only four GMI-sessions with the team, due to the restricted time available of both teamleaders and employees. A more extensive program was not feasible in the work setting, as the GMI-sessions were given during work hours and supervisors were reluctant in spending more hours on this intervention.

Although many statistical tests were performed, we did not perform an adjustment for multiple testing. The reason for not doing this was that we were not interested in single significant results, but in the broader picture. Because of multiple testing, single significant results should be interpreted with caution.

Finally, in the current study, multilevel analyses were applied using the maximum likelihood estimation procedure to account for missing data. The results did not show that certain predictor variables were related to missing data and we assumed that missing's were at random (MAR). Maximum likelihood allows all available data to be used and for this imputation of missing data is considered unnecessary. The maximum likelihood estimate of a parameter is the value of the parameter that is most likely to have resulted in the observed data [78]. It has been revealed that using multilevel analysis for an incomplete dataset is better than applying imputation methods [78], [79]. With imputation, multivariate regression techniques are used to predict missing values on the basis of observed factors and several imputed datasets are created to account for uncertainty. The difference between the two is that for maximum likelihood no separate model is created, and there is no difference between the imputation model and the analysis model. With the multiple imputation technique, imputation is done separately from the analyses. Every time you apply it to a given set of data, one will get slightly different parameter estimates and test statistics. Consequently, researchers applying the same methods to the same data could reach different results. While maximum likelihood always produces the same results for the same set of data. Altogether, we are not able to state which method for handling missing data is outstanding as neither approach is inherently better than the other. Therefore, we encourage further statistical research to examine the methods of treating missing data in multilevel analyses.

### Research and practical implications

We believe that the combined social and physical environment intervention has the potential to improve the need for recovery. The present study consisted of a general healthy and well-functioning population, which makes it hard to have a large impact on improving the need for recovery. It is therefore recommended to implement the social and physical environment intervention among a population with higher baseline values on need for recovery. For improving the intervention, we also would suggest to provide a physical activity opportunity in addition to the GMI, for example, organize lunch walking or yoga classes at work.

Further, integration of the social media platform by means of designing a strategic plan, incentives for regular use (promotion codes for free workshops/trainings, to do a free tests/get advice on a healthy lifestyle, assign for a competition) and linking to other platforms such as facebook is recommended. Additionally, it is recommended to implement more drastic physical interventions (i.e., restructuring of entire department floor), because the relatively ‘simple’ environment modifications that were used (e.g., placing signs to promote stair use) did not result in changes in need for recovery.

The present study showed that employees and supervisors rated support by the financial service provider and their managers as relatively low. Many organizations are still not convinced that worksite health promotion programs offer advantages (e.g., reducing risk factors, absenteeism and improving performance) for their workforce [80]. Instead, they tend to think that these programs distract people too much from their daily work-tasks [81]. Attention should be paid to improve support levels as previous research showed that commitment of higher management is of utmost importance for successful interventions; ‘board on board’ is needed [82]–[84].

The strongest non-significant reduction for need for recovery was found in the combined intervention group, which is in line with the socio-ecological approach [28]. For the other outcomes, the combined intervention did not result in the strongest effects and hardly any overlap in effects was found with the separate social and physical environment intervention. Further, the interventions mainly seemed to have an effect on physical activity related outcomes (i.e., stair climbing, active commuting, sedentary behavior at work, and leisure activities). Possibly, the participating financial service provider supported physical activity more as they had a recently opened company fitness centre. Also, attention should be paid to a reliable, valid and responsive questionnaire for measuring detachment and relaxation. Another recommendation is that objective measurements should be added to assess small breaks at work, stair climbing at work, physical activity and sedentary behavior at work, because self-reports are subject to measurement error.

### Conclusion

Strong conclusions about the combined intervention's effectiveness in improving the need for recovery cannot be made, as we did not find significant effects of our interventions on need for recovery. The present study consisted of a general healthy and well-functioning population, with relatively low and a beneficial need for recovery, which makes it hard to improve the need for recovery any further. It is therefore recommended to implement the social and physical intervention among a population with higher baseline values of need for recovery. Furthermore, the intervention itself could be improved by increasing the intensity of the intervention (for example weekly GMI-sessions), providing physical activity opportunities and exercise schemes, and by more drastic environment interventions (restructuring entire department floor).

## Acknowledgments

We would like to thank all teamleaders and employees who participated in this study. The authors also wish to thank Robine van der Starre and Ruben Kraaijeveld for their help with collecting the data.

## Supporting Information

S1 Checklist (DOC)

S1 Protocol (DOCX)
