# Supplementary material for: Effectiveness of a Worksite Social & Physical Environment Intervention on Need for Recovery, Physical Activity and Relaxation; Results of a Randomized Controlled Trial
Source: PLoS One. 2014 Dec 26;9(12):e114860. doi: 10.1371/journal.pone.0114860 (PMC4277283; doi:10.1371/journal.pone.0114860)
Supplement: S1 Protocol — (DOCX) [file pone.0114860.s002.docx]

**Research protocol approved by ethics committee VUmc**


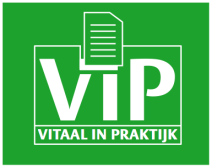


Evaluation of an intervention to decrease the need for recovery in office employees by increasing daily physical activity and relaxation at work

Jennifer Coffeng, MSc.

EMGO+ Institute for Health and Care Research

Department of Public and Occupational Health

VU University Medical Center

Van der Boechorststraat 7

1081 BT Amsterdam

T: 020-4446137

E: j.coffeng@vumc.nl

W: [www.vitaalinpraktijk.nl](http://www.vitaalinpraktijk.nl)

| **Protocol ID** | NTR2553 (Nederlands Trial Register) |
| --- | --- |
| **Short title** | VIP in Insurance |
| **Version** | 1 |
| **Date** |  |
| **Promotor** | W. van Mechelen, MD, PhD, FACSM, FECSS.  Department of Public and Occupational Health  EMGO+ Institute for Health and Care Research  VU University Medical Center  Van der Boechorststraat 7  1081 BT Amsterdam, the Netherlands |
| **Co-promotor, daily supervisor** | S.F.A. Duijts, PhD.  Department of Public and Occupational Health  EMGO+ Institute for Health and Care Research  VU University Medical Center  Van der Boechorststraat 7  1081 BT Amsterdam, the Netherlands |
| **Co-promotor** | I.J.M. Hendriksen, PhD.  TNO Expertisecentrum Life Style  Wassenaarseweg 56, Postbus 2215,  2301 CE Leiden, the Netherlands |
| **Co-promotor** | C.R.L. Boot, PhD.  Department of Public and Occupational Health  EMGO+ Institute for Health and Care Research  VU University Medical Center  Van der Boechorststraat 7  1081 BT Amsterdam, the Netherlands |
| **Principal investigator** | J.K. Coffeng, MSc.  Department of Public and Occupational Health  EMGO+ Institute for Health and Care Research  VU University Medical Center  Van der Boechorststraat 7  1081 BT Amsterdam, the Netherlands |
| **Sponsor (in Dutch: verrichter/opdrachtgever)** | Delta Lloyd Groep Zorgverzekeringen B.V. |
| **Independent physician** | P.M.M. Gallee, MD.  Arbeid & Milieu Dienst  VU University Medical Center  Van der Boechorststraat 1  1081 HV Amsterdam, the Netherlands |

**PROTOCOL SIGNATURE SHEET**

| **Name** | **Signature** | **Date** |
| --- | --- | --- |
| Prof. dr. Willem van Mechelen  Head of department/Promotor |  |  |
| Dr. Saskia Duijts  Co-promoter |  |  |
| Drs. Jennifer Coffeng  Principal investigator |  |  |

#

Table of contents

[1. Introduction 13](#_Toc292827142)

[1.2. Development of the VIP program 14](#_Toc292827143)

[2. Objectives and research questions: VIP in Insurance project 17](#_Toc292827144)

[3. Methods 18](#_Toc292827145)

[3.1 Population 18](#_Toc292827146)

[3.2 Inclusion and exclusion criteria 18](#_Toc292827147)

[3.3 Design 18](#_Toc292827148)

[3.4 Study procedures 18](#_Toc292827149)

[3.5 Sample size calculation 19](#_Toc292827150)

[4. Study parameters/endpoints 19](#_Toc292827151)

[4.1 Primary outcome measure 20](#_Toc292827152)

[4.2 Secondary outcome measures 20](#_Toc292827153)

[4.3 Other measures 21](#_Toc292827154)

[4.4 Interventions within the VIP program 22](#_Toc292827155)

[4.4.1 (A). Group motivational interviewing 22](#_Toc292827156)

[4.4.2 (B). Environmental modifications 24](#_Toc292827157)

[4.5 Withdrawal of individual subjects 25](#_Toc292827158)

[4.5.1 Specific criteria for withdrawal 25](#_Toc292827159)

[4.6 Replacement of individual subjects after withdrawal 25](#_Toc292827160)

[4.7 Follow-up of subjects withdrawn from treatment 25](#_Toc292827161)

[4.8 Premature termination of the study 25](#_Toc292827162)

[5. Safety reporting 25](#_Toc292827163)

[5.1 Section 10 WMO event 25](#_Toc292827164)

[5.2 Adverse and serious adverse events 25](#_Toc292827165)

[5.3 Follow-up of adverse events 25](#_Toc292827166)

[5.4 Data Safety Monitoring Board (DSMB) 25](#_Toc292827167)

[6. Statistical analysis 26](#_Toc292827168)

[6.1 Economic evaluation 26](#_Toc292827169)

[6.2 Process evaluation 27](#_Toc292827170)

[7. Ethical considerations 27](#_Toc292827171)

[7.1 Regulation statement 27](#_Toc292827172)

[7.2 Objection by minors or incapacitated subjects 27](#_Toc292827173)

[7.3 Benefits and risk assessment, group relatedness 27](#_Toc292827174)

[7.4 Compensation for injury 28](#_Toc292827175)

[7.5 Incentives 28](#_Toc292827176)

[8. Administrative aspects and publication 28](#_Toc292827177)

[8.1 Handling and storage of data and documents 28](#_Toc292827178)

[8.2 Data entrance 28](#_Toc292827179)

[8.3 Privacy concerns 28](#_Toc292827180)

[8.4 Data storage 28](#_Toc292827181)

[8.5 Amendments 29](#_Toc292827182)

[8.6 Annual progress report 29](#_Toc292827183)

[8.7 End of study report 29](#_Toc292827184)

[8.8 Public disclosure and publication policy 29](#_Toc292827185)

[Appendix 1: Design of the VIP project 30](#_Toc292827186)

[Appendix 2: Flow Diagram of the VIP project 31](#_Toc292827187)

[Appendix 3: Timeline of the VIP project 32](#_Toc292827188)

#

**SUMMARY**

**Rationale:** The ‘VIP (Vitality in Practice) in Insurance’ project is situated at a financial service provider in the Netherlands. This employer has great ambitions regarding the health of its employees and has already taken steps towards offering options to improve employees’ wellbeing. The management of the financial service provider identified low physical activity and relaxation levels, and high need for recovery, in its employees. Within the ‘VIP in Insurance’ program, an intervention was developed, in close cooperation with several representatives from the financial service provider, to decrease the need for recovery, by improving daily physical activity and relaxation behaviour at work.

**Objective:** The aim of this study is to evaluate an intervention program consisting of group motivational interviewing (GMI), supported by a social media platform, and/or environmental modifications, to decrease the need for recovery in office employees by increasing daily physical activity and relaxation levels.

**Study population:** The research population will consist of office employees of a financial service provider in the Netherlands.

**Study design:** In this study, a randomized controlled trial design will be employed, with pre-stratification on environmental modifications. At several floors of one of the main buildings of the financial service provider, two strata will be created with (6 wings) and without (7 wings) environmental modifications. Within each stratum, wings will be randomly assigned to GMI or no GMI. Randomisation will take place at wing level. The VIP project will consist of four research arms, i.e. (1) both GMI and environmental modifications, (2) environmental modifications/no GMI, (3) GMI/no environmental modifications, (4) no GMI/no environmental modifications (control group). Questionnaire data will be gathered at baseline (T0), at 6 months (T1), and at 12 months (T2) follow-up, in both employees and their teamleaders. In addition, cost-effectiveness and cost-utility analyses will be conducted from a societal and company perspective, and a process evaluation will be performed.

**Interventions**: (A). GMI intervention: teamleaders of the financial service will be trained to become a GMI-coach. After the two-day training, they will lead a GMI-group, consisting of their own employees. GMI will be applied for 3 sessions of 90 minutes each, over a period of 6 weeks, and will end with a follow-up meeting of 90 minutes, 2 months after the final session. The GMI groups will be supported by a social media platform. Employees will be stimulated through this platform to join physical and relaxation activity groups. (B). Environmental modifications: changes in the physical environment will be made to facilitate daily physical activity and relaxation at work, such as redesigning the coffee corners and the meeting rooms. The control group will receive no specific interventions and will proceed working as usual. A special VIP page will be created on the intranet for all employees, summing up the current offers of the financial service provider which are related to stimulating daily physical activity and relaxation. Furthermore, every two months, all employees will receive a newsletter about the VIP project.

**Main study parameters/endpoints:** Primary outcome measure of the study is need for recovery. Secondary outcome measures are daily physical activity, sedentary behaviour and relaxation/detachment. Furthermore, general health and work-related factors, such as exhaustion, sleep quality, stress, productivity and absenteeism will be measured. In addition, a subsample of 100 employees will be asked to wear an accelerometer for one week during the three measurement periods to measure sedentary and daily physical activity behaviour objectively.

**Hypothesis/anticipated results:** We hypothesize that the VIP program will significantly lower the need for recovery in office employees by increasing daily physical activity and relaxation, compared to the control group, both at short-term (6 months) and long-term (12 months) follow-up.

# 1. Introduction

In the past decades, the pressure of work has substantially increased. More than sixty percent of the employees in the EU experience that they are ‘working to tight deadlines’ (1). Working under pressure or working with high job demands has a strong negative impact on employees’ health and well-being, especially where employees have little decision latitude or little support from colleagues and/or managers. Employees with these unfavourable job characteristics are at risk to experience health problems, such as cardiovascular diseases (2), low back and neck pain (3) and psychological distress (4). Multiple work-related health problems seem to be preceded by a higher need for recovery (2;5). Need for recovery from work can therefore be considered as an important indicator for employees who are at risk for work related health problems (2;5).

The concept of need for recovery was deduced from the effort-recuperation model (6). It refers to the extent that the work task induces a need to recuperate from work induced effort. Need for recovery is characterised by temporary feelings of overload, irritability, social withdrawal, lack of energy and reduced performance (7). When time is lacking for recovery, initial load reactions are likely to accumulate, resulting in increased blood pressure (8), sleeping problems, (9) fatigue (10;11) and cardiovascular diseases (2).

For organisations, it seems worthwhile to invest in intervention programs that prevent higher need for recovery among their employees. Employees should pursue activities during work to recover. Detachment from work can be helpful for recovery. Psychological detachment is defined as the employee’s feeling to be mentally and/or physically away from work-related activities (12). Detachment has been identified to be particularly difficult when one has to deal with high job demands (7;13;14).

Earlier studies showed that breaks during work are needed to restore personal resources for future job demands (15). A break during work can be, for example, taking a walk or having coffee with colleagues. Engaging in breaks has been associated with positive affect and lower levels of negative feelings (15;16). Engaging in work-related tasks during breaks, and the resource depletion that goes along with it, should be avoided (17). Psychological detachment created during breaks is related to and reduces the need for recovery (12;14). When having insufficient time to detach from work, both emotional exhaustion (18;19) and the need for recovery (18) will increase. Following this line of reasoning, it is vital for organizations that employees regularly detach from work during the day.

It is hypothesized that physical activity and relaxation are useful strategies to improve detachment from work and lower the need for recovery from work. Previous studies showed that physical activity and relaxation were effective in altering physiological symptoms, somatic symptoms and psychological variables of stress (20). Physical activity programs are demonstrated to reduce work stress (21), absenteeism (22;23) and to improve job satisfaction (24). Relaxation helps to reduce blood pressure (25), heart rate and other symptoms of tension (26). It was shown that relaxation improves a positive mood (27), reduces anxiety, improves self-esteem and personal relationships (28). It can be concluded that both physical activity and relaxation can be useful in organizations for reducing employees' need for recovery from work.

# 1.2. Development of the VIP program

The VIP program was developed, using elements of the Intervention Mapping protocol (29). In this stepwise approach, a blueprint for the intervention was developed in close cooperation with the target group, combining scientific evidence with practice-based information. The management of the financial service provider indicated that improving daily physical activity and relaxation should be the main starting points for intervention development. Using interviews and questionnaires, we collected information on the employee’s attitude towards daily physical activity and relaxation. During these interviews, employees expressed that they generally have low daily physical activity and relaxation levels. Also, it was shown that they have a high need for recovery. Derived from the process of Intervention Mapping, two objectives for the VIP program were chosen: (1) office employees should be stimulated to increase their levels of daily physical activity by increasing active home-to-work transport and physical activity at work; (2) office employees should be stimulated to increase relaxation by increasing regular detachment during work hours. For developing the program, it was important to specify determinants for daily physical activity and relaxation. Based on the interviews, the most important determinants of daily physical activity and relaxation were: awareness, knowledge, attitude, subjective norm, self-efficacy and physical environment. Based on these determinants, methods and strategies were chosen. To specify the intervention program, tools and materials were selected to support the strategy. Decisions about suitable methods, strategies, tools and materials were made together with the project group and feedback from experts (Table 1). Subsequently, two components of the VIP program were chosen, i.e. (A) Group Motivational Interviewing (GMI), supported by a social media platform and (B) environmental modifications.

Table 1. Methods and Strategies of the VIP project

| **Determinant** | **Theoretical method** | **Practical strategy** | **Tools and materials** |
| --- | --- | --- | --- |
| *Awareness* | Self-monitoring | Monitoring of own behaviour | Dairy in workbook. Self complete logs to monitor own physical activity and relaxation behaviour in last week. |
| *Knowledge* | Passive learning/providing information | Provide written and verbal information | Promote current facilities of financial service provider on social media platform/intranet/ and give information during Group Motivational Interviewing sessions. |
| *Attitude* | Active processing of information/active learning | Brainstorming about barriers/Group discussion | A group discussion about barriers during Group Motivational Interviewing sessions. |
| *Subjective norm* | Mobilizing social support | Invite employees to formulate who can provide support | During a Group Motivational Interviewing session: discussion about who can provide support.  +  Employees can join or create physical activity and relaxation groups on the social media platform.  + Forum on social media platform with exchanging tips on daily physical activity/relaxation. |
| *Self efficacy* | Reinforcement  Goal setting | Provide positive feedback on changes in self efficacy: evaluation of changes  Facilitate formulation of goals | During Group Motivational Interviewing positive feedback will be given by teamleader and employees. Also new arguments to cope with difficult situations is given.  During Group Motivational Interviewing: Worksheets to help extract planning goals (when, where, with whom?). Discussion about barriers/ difficult situations and possible solutions. |
| *Physical environment* | Environmental changes | Change work environment | Ask for interior advice. Change colours/ lighting/ desks etc. |

(A) Group Motivational Interviewing (GMI)

Over the last years, the technique of individual Motivational Interviewing (MI) has gained strong empirical support in work settings (30-34). MI in work settings was implemented to improve health- related behaviours, such as physical activity, dietary behaviour, mental health, and to diminish substance abuse. There is increasing support that individual MI can be adjusted to a group format (35-40). Group motivational interviewing (GMI) uses the concepts of self-determination theory (41) and individual motivational interviewing (42). The essence of GMI is to apply the individual MI concepts to a group setting. A group setting has several benefits, i.e. sharing experiences, providing feedback and giving support (40). GMI helps to create an autonomous supportive environment, in which behaviour change is encouraged (36). Evidence for the beneficial effects of groups in work settings can be found in research on group education. For instance, an 8-week nutrition education group program developed for university employees, showed increased nutrition knowledge, reduced calorie intake and cholesterol intake (43). Another study of a 9-week group education program on diet and physical activity with university staff showed reduced lipid, glucose and insulin levels (44). Given that individual MI and group education programs have shown to be effective in work settings, it can be assumed that GMI in the work setting might be effective for lowering the need for recovery by improving daily physical activity and relaxation behaviour too. Supporting the GMI by a social media platform will help to improve effective communication, staying informed on news topics, finding answers and receiving help (45).

(B) Environmental modifications To date, most environmental modifications to improve a healthy lifestyle focused on healthy nutrition and improving physical activity (46). Examples are food labelling, changes in food offering, providing exercise space and exercise equipment, red line routes to promote lunch walking, posters to promote stair use and a walking track (46). Research has shown that changing work areas (i.e. office layout and/or office furniture) could positively influences employee's attitudes (47), job satisfaction and performance (48-51). For example, Vink et al. (52) asserted that redesigning the coffee corner, e.g. placing informal seats, will increase the number of conversations at the coffee corner. They found that these conversations were 4 out of 5 times about work and were beneficial to the employees’ productivity and creativity (52). Furthermore, in the study of Cohen et al. (53), it was demonstrated that the design of a meeting room, but also the lighting, noise and seating, may have impact on the quality of the meeting. Leach et al. (54) proved that environmental modifications to a meeting room will improve comfort for the employee and helps the employee to focus on the meeting. Other examples of effective interventions were placing stand-up tables, i.e. a previous study showed that stand-up meetings are faster than sit-down meetings (55); painting office walls, i.e. it has been discovered that this may positively influence productivity levels (56). Specifically, working in a red environment increases productivity, but it is more distracting than working in a white environment (57). A blue-green work environment is seen as more relaxing and is preferred by employees. On the negative side, a red colour reduces arousal levels and consequently productivity (57). Next, adjustment of lighting. That is, in the study of Veitch (48), employees worked under six different lighting conditions a day. When different lighting was applied, they were in a better mood and scored higher on well-being. Finally, modifications to decrease office noise (58). Concentration is reduced by telephone ringing and people talking in the background (59). Hearing problems during the day increases the need for recovery at the end of the day (60). Studies showed that sound-masking, placing indoor plants or indoor vegetation in the work environment are really appreciated (61) and increase a positive mood and performance (62).

# 2. Objectives and research questions: VIP in Insurance project

The primary objective of this study is to evaluate the effectiveness of the ‘VIP (Vitality in Practice) in Insurance’ program, i.e. GMI, supported by a social media platform, and/or environmental modifications, on the need for recovery, daily physical activity and relaxation levels in office employees. Secondary objectives are to evaluate the cost-effectiveness and cost-utility of the VIP program and to evaluate the process of implementation of the VIP program.

We hypothesize that the VIP program will significantly lower the need for recovery in office employees by increasing daily physical activity and relaxation, compared to the control group, both at short-term (6 months) and long-term (12 months) follow-up. We expect that the intervention arm with both the GMI-intervention and the environmental modifications will be more successful to lower the need for recovery in office employees, than the intervention arms with only GMI or only environmental modifications.

Research questions

The research questions of the ‘VIP in Insurance’ project are:

1) What are the effects of the VIP program on need for recovery in office workers?

2) What are the effects of the VIP program on daily physical activity and relaxation in office workers?

3) What are the cost-effectiveness and cost-utility of the VIP program from a societal and company perspective?

4) What are the strengths and weaknesses of the VIP program when implementing the program?

# 3. Methods

# 3.1 Population

The study sample consists of office employees (18-64 years) of a financial service provider in the Netherlands. The building of this financial service provider has 10 departments with 24 wings. Each wing has the capacity for 6 teams, corresponding with a total of 60-80 employees per wing. Wings on a department level are separated from one another. In the present study, teamleaders and their employees of 13 wings will be invited to participate. We decided to exclude 4 departments (11 wings) beforehand, since environmental modifications are already ongoing or planned in these departments.

#

# 3.2 Inclusion and exclusion criteria

Employees are eligible for participation in the study, when they are able to complete a Dutch (online) questionnaire and when they are expected to be employed at the financial service provider during the complete follow-up period. Employees will be excluded from the study, if they are on long-term sick leave (≥ 4 weeks) at baseline.

# 3.3 Design

In this study, a randomized controlled trial design will be employed, with pre-stratification on environmental modifications. That is, two strata will be created, with (6 wings) and without (7 wings) environmental modifications. Within each stratum, wings will be randomly assigned to GMI or no GMI. Randomisation will take place at wing level. We decided to apply cluster randomisation on wing level to avoid contamination with employees in the control group. Consequently, the study will consist of four research arms, i.e. (1) both GMI and the environmental modifications, (2) environmental modifications/no GMI, (3) GMI/no environmental modifications, (4) no GMI/no environmental modifications (control group). The design of the VIP project is shown in appendix 1.

# 3.4 Study procedures

Managers and directors of all potentially participating wings will be invited by email for a short VIP kick-off meeting. During this kick-off meeting, information about the VIP program will be given to the managers/directors and they will be asked to stimulate participation of their teamleaders and employees. Subsequently, all teamleaders (N=80) from 13 wings will be invited by email for an exclusive VIP kick-off meeting for teamleaders (approximately 45 minutes). During this meeting, information about the VIP program will be given and teamleaders will be asked to stimulate their employees to participate in the VIP program. Directly after this meeting, they will receive two envelopes: one with a letter and informed consent form, for themselves, and the other including all letters, informed consent forms and flyers for their employees. Employees have to give their consent for participation in the project and for obtaining data on sick leave. Data on sick leave and the duration of employment will be obtained from the HRM department, only when the employee has given his/her consent. After one week, the VIP researcher will personally collect all informed consent forms, both from the teamleader him/herself and his/her employees. In case of no response, a reminder will be sent to the teamleaders and the VIP researcher will visit them personally. Employees will be asked to give their email address on the informed consent form, which will be used for sending the online baseline questionnaire. It will be emphasized that they can only participate, when both the teamleader and more than half of the employees within a team have given their consent. Furthermore, a wing is only eligible for randomisation, when more than half of the teams within a wing have given their consent. When teamleaders and their employees are excluded from the VIP program, they will be informed by an email of the VIP researcher. A flow diagram of the VIP project is shown in appendix 2.

Randomisation of GMI will be performed after the baseline measurement (T0) and will be prepared and executed by an independent researcher, by using a computer generated list from SPSS. After baseline, extensive (online) questionnaire data will be gathered at 6 months (T1), and at 12 months (T2) follow-up, in both the intervention and the control group. At 3, 6, 9 and 12 months follow-up, data is gathered for the economic evaluation. At 6 and 12 months these questions will be added to the extensive online questionnaire. At 3 and 9 months these questions will be asked separately by a small online questionnaire. Furthermore, at 6 months follow-up (at the end of the intervention) questions to evaluate the program will be added. Reminders for the questionnaires will be sent, after one week. A timeline of the VIP project can be found in Appendix 3.

3.5 Sample size calculation The sample size calculation is based on the main outcome measure, i.e. need for recovery, measured by the need for recovery questionnaire (63). The mean need for recovery score is 27,30 (SD=29,75) according to the validation study of Van Veldhoven et al. (63). Minimal relevant difference on the need for recovery scale, with a range from 0 to 100, was set on 12. This was based on the articles by de Croon et al. (64) and Kuijer et al. (65), in which on average a relevant difference of 12 was found in nurses, truck drivers and refuse collectors, after an intervention, such as change in work hours (64) or job rotation (65). Using a power of 0.9, in total 260 employees are needed. Because randomisation takes place at wing level, a cluster correction was applied. An intraclass correlation (ICC) of about 0.025 is assumed, based on previous studies showing that worksite level ICC’s for health-related outcomes are generally small (66-68). This leads to a cluster correction factor of 1 + (13-1)* 0.025 = 1.3. Based on this cluster correction, a total of 1.3*260 = 338 employees will be needed. Furthermore, by applying a correction for repeated measurement we will win some precision, therefore an additional correction by a factor 0.9 seems justified, resulting in 0.9×338=305 employees in total. Finally, accounting for a loss to follow-up of 25% in twelve months, a total of 404 employees for the four intervention arms will be needed at baseline. Based on the source population of 980 employees (13 wings), the number of 404 employees is reached at a participation rate of 42 percent. This percentage is considered feasible, because all teamleaders and their employees within a wing will be asked to participate.

# 4. Study parameters/endpoints

The primary outcome measure is need for recovery. Secondary outcome measures are physical activity, sedentary behaviour and relaxation at/detachment from work. Furthermore, several demographics (i.e. age, gender, education), work and health-related factors will be examined. A subsample will be invited to wear an accelerometer.

# 4.1 Primary outcome measure

Need for recovery will be assessed using the need for recovery after work scale of the Dutch VBBA questionnaire (63). This Dutch version of the Questionnaire on the Experience and Evaluation of Work (Dutch abbreviation VBBA) consists of eleven dichotomous items (yes/no). The VBBA has been evaluated among 601 employees from various organisations in the Netherlands, and showed satisfactory validity (r=0.65) and reliability (r=0.87) (63).

#

# 4.2 Secondary outcome measures

Daily physical activity will be assessed by the Short Questionnaire to Assess Health Enhancing Physical Activity (SQUASH) (69). This questionnaire assesses the duration, frequency, intensity of active home-to-work transport, physical activity at work, sedentary time at work and home, activity in leisure time and household activities, and sport. The SQUASH is a fairly reliable (r=0.58) and reasonably valid (r=0.45) questionnaire. Its validity and reliability are comparable to other physical activity questionnaires (70).

Physical activity and sedentary behaviour will also be measured objectively in a random sample of 100 participants, consisting of the GMI group (n=25), the GMI and environmental modifications group (n=25), the environmental modifications only group (n=25) and the control group (n=25). This subsample will be asked to wear the accelerometer during a period of 7 days, both at baseline, 6 and 12 months follow-up. On all days of the week, participants fill out a short questionnaire (diary) on the exact wearing times of the Actigraph (i.e. the time at which the Actigraph was put on and off) and the times of leaving and arrival at home and work (to determine the exact wearing time at work and leisure time). At the end of each week, they will be asked to hand it back to the researcher/research assistant on appointment.

Detachment and relaxation will be assessed by the recovery experience questionnaire, which is developed by Sonnentag et al. (71) and showed satisfactory reliability (r=0.84) and reasonable validity (r=0.46). A Dutch version of this questionnaire showed high reliability (r=0.92) in a pilot study by Bloom et al. (72). We will adapt this scale to a work context, starting each item with “During a break at work…”, instead of “During time after work…” as written in the original questionnaire of Sonnentag et al. (71). This detachment and relaxation questionnaire consist of 8 items and is derived from the recovery experience scale (71). It consists of items such as “I forget about work”, “I don’t think about work at all”, “I kick back and relax” and “I do relaxing things”. Each item can be scored on a 7-point scale, ranging from “never” to “always”. Both the original and modified version of the recovery experience scale (71) will be assessed.

# 4.3 Other measures

Baseline measurements will include demographic questions, such as age, sex and education level. Other measures that will be asked at baseline, 6 months (T1), and at 12 months (T2) follow-up are described below.

Exhaustion will be assessed by means of a subscale of the Oldenburg Burnout Inventory (OLBI) (73). The OLBI consists of 8 items about exhaustion and disengagement and can be scored on a 4-point scale, ranging from “totally agree” to “totally not agree”. It showed reasonable validity (r=0.52) and satisfactory reliability (r=0.80) in different occupational groups (73).

General health and mental health will be measured by items of Dutch validated version of the Rand-36 (74), which showed satisfactory reliability (r=0.83) and reasonable validity (r=0.49). General health perceptions are measured by asking employees to give an indication on how they perceive their health (on a 5-point scale ranging from "excellent" to "bad") and to indicate on four propositions (e.g. “I fall ill more easily than others”) to which extent they agree "totally" or "totally not agree", on a 5-point scale. To assess mental health, employees will be asked to indicate on a 6-point scale ("always" to "never") how often they felt full of life, worn out, tired and full of energy, during the past four weeks (74).

To measure stress, the short form of the perceived stress scale (PSS; 4 items; 5-point scale "never" to "very often") will be used (75). PSS-4 is considered to be sound, but it has a rather low internal reliability (r=0.60). Both the 10- and 14-item self-report of PSS have higher satisfactory reliability and validity (75). Still, the PSS-4 was chosen for this study because it is not meant as a diagnostic instrument. It will only be used to make comparisons between past and current stress levels.

Sleep quality will be assessed by the Jenkins sleep problems scale. (72) This scale contains 4 items, i.e. trouble falling asleep, continue sleeping, waking up several times at night and waking up feeling tired and worn out. The scale showed an acceptable test-retest reliability (r=0.59) and internal consistency of (r=0.63).

Determinants of behaviour and behaviour change for physical activity and relaxation will be examined by means of the lifestyle behaviour questionnaire (DLBQ), which is validated by Lakerveld et al. (76). Determinants of interest for daily physical activity and relaxation are awareness, knowledge, attitude, subjective norm, self-efficacy, physical environment. The items are measured on a 5-point scale from “totally agree” to “totally not agree” (77;78). A study of Lakerveld et al. demonstrated that the DLBQ is able to measure determinants that precede the intention to change physical activity in adults (73).

Absenteeism data will be collected by questionnaires at 3, 6, 9 and 12 month’s follow-up through self-report, as well as by company registrations. The World Health Organization Health and Work Performance Questionnaire (WHO-HPQ), a self-report measure concerning absenteeism and presenteeism, will be used (79). To illustrate, participants will be asked to give "the number of days missed due to mental or physical health problems, during the last 4 weeks".

Work performance will be assessed with the Individual Work Performance Questionnaire (IWPQ), (80). The IWPQ consists of 20-30 questions in four subscales: task performance, contextual performance, adaptive performance, and counterproductive work behaviour. Answers can be scored on a 7-point scale, ranging from “never” to “always”. The IWPQ is a newly developed instrument based on a review of the work performance literature, existing work performance questionnaires, and expert opinions (80).

Job characteristics will be assessed with 30 items (4-point scale from "totally agree" to "totally not agree") of the validated Dutch version of the Job Content Questionnaire (JCQ), (81) i.e. job demands, skill discretion, decision authority, supervisor and co-employee support and job insecurity. It showed satisfactory reliability (r=0.72). The JCQ is widely used (81) and derived from the well-known Job Demands-Control-Support model of Karasek and Theorell (82).

Work engagement will be measured, using the validated Utrecht Work Engagement scale (UWES ), which assesses vitality (6 items), dedication (5 items) and absorption (6 items) (79). Each item can be scored on a 7-point scale, ranging from “never” to “always”. The concepts vitality, dedication and absorption will give an indication about employees' engagement level (79). The psychometric properties of this questionnaire have been tested and results indicate an acceptable internal consistency of vitality (r=0.68-0.80), dedication (r=0.91) and absorption (r=0.73-0.75) (79).

# ****4.4 Interventions within the VIP program****

The ‘VIP in Insurance’ program encompasses two elements, i.e. (A) Group Motivational Interviewing (GMI), supported by a social media platform, and (B) environmental modifications. The control group will receive no specific interventions and will proceed working as usual. A special VIP page will be created on the intranet for all employees, summing up the current offers of the financial service provider which are related to daily physical activity and relaxation. Furthermore, every two months, all employees will receive a newsletter about the VIP project.

#

# 4.4.1 (A). Group motivational interviewing

In this project, teamleaders will be trained to supervise the GMI-sessions, instead of professionals. The presence of GMI-skills within the company will provide long-term benefits. The teamleaders of the wings allocated to the GMI-intervention will be invited for the two-days training in GMI, which will be conducted by a GMI-professional. Acquired knowledge can be applied to other areas, e.g. yearly assessments of employees. After completing the 2-days training, the VIP-teamleaders will directly start with the GMI-sessions in their team. The teamleaders will conduct three GMI-sessions of 90 minutes each, within a period of 6 weeks (every 3 weeks a session). A booster session will be scheduled, 2 months after the final session. All sessions will take place during work hours. The main aim of all these sessions is to stimulate daily physical activity and relaxation. The key elements of the sessions are to develop discrepancy, roll with resistance, express empathy and support self-efficacy (42). During the GMI-sessions, the team leader will focus on fostering a readiness to change, lowering resistance and ambivalence towards changing daily physical activity and relaxation behaviour. For each session, materials will be developed. There is a manual, which consists of theoretical information and detailed instructions. The VIP-teamleader will supervise the sessions by following the instructions in the manual. Furthermore, when the VIP program is ongoing, the VIP-teamleaders will have two meetings of 90 minutes each, supervised by a GMI-professional, to share experiences with each other.

Session 1 “launch of GMI”

Session 1 will focus on welcoming the employees and creating a warm and trustworthy atmosphere. The agenda of the upcoming GMI-sessions will be explained in this session. Furthermore, the social media platform will be introduced and a competition with a step counter will be explained. Specifically, the employees will receive a step counter and they will be stimulated to raise their steps with 10 percent each week. It is a virtual team competition from Amsterdam to Paris. Also, the employees will be informed on the main subjects of the program, i.e. daily physical activity and relaxation. After this introduction, the employees will be asked to interview each other in pairs on their current physical activity and relaxation behaviour at work. Subsequently, the employees will be asked to fill in a handout, i.e. a timeline of their current physical activity and relaxation behaviour. In addition, they will interview each other in small groups about possible difficulties that could occur when changing their current lifestyle. At the end, homework is given by the GMI-teamleader.

Session 2 “considering physical activity and relaxation behaviour” First of all, the state of the virtual competition from Amsterdam to Paris will be briefly discussed. Then, the employees will fill in a handout about importance, trust and ability to change their physical activity and relaxation behaviour. In pairs, they will discuss their findings. Furthermore, the stages of change concept (83) will be explained by the GMI-team leader and the developed wheel of change map will be showed. Here, employees will mark their stage of change with a chip. Subsequently, employees will be asked to reflect on their own stage, during a group discussion, and to describe how they could change their behaviour. The last assignment of this session is to fill in a hand-out about desire, ability, results and needs. They will be asked to discuss this in pairs and to share their results in the group. Again, homework will be given.

Session 3 “action”

Main aim of this session is to formulate action plans to improve daily physical activity and relaxation. First of all, by means of the wheel of change map, employees will be asked to place their chip on their current position. The results will be discussed in the group. The GMI-team leader will inform the group about the financial service provider's vitality program and the facilities they can use. Then, individual goals will be formulated about improving daily physical activity and relaxation. In addition, a handout about successful change will be filled in and discussed in pairs. After stating the goals, and referring to former success stories, an individual action plan to improve daily physical activity and relaxation will be formulated. This action plan will be discussed in pairs. Homework for the upcoming weeks will be given, such as to follow the individual action plan.

Booster session

After two months, a booster session is scheduled. Again, by means of the wheel of change map, employees will be asked to place their chip on their current position. The outcomes will be discussed and solutions will be introduced by sharing experiences. During a group discussion, employees are asked to evaluate the last two months. Furthermore, employees will be asked to critically evaluate previous sessions by examining formerly filled in handouts. If necessary, the action plan or other hand-outs can be revised. Subsequently, employees will be asked to give each other compliments non-verbally, by means of a handwritten card. The employees will be stimulated by the GMI-team leader to remain improving their daily physical activity and relaxation behaviour. The booster session will end by evaluating the VIP program.

Supporting social media platform

During the GMI-sessions, a social media platform will be available through an external website. The VIP social media platform is meant for both employees and teamleaders, allocated to the GMI-intervention arms of the study. The VIP researcher will send a login-code by email. The main goal of this platform is that employees will create or join physical activity and relaxation groups. Potential activities can be playing on a dartboard, lunch walking, doing physical exercise and taking part in a book club. Employees can create their own groups or they can join other groups who are created by their colleagues. The teamleader can be invited to join the employees on the platform. Furthermore, the employees can find appealing information, which will be continually updated by the researcher of the VIP project. For the teamleaders, there is a separate group on the social media platform where they can share experiences with other teamleaders.

# 4.4.2 (B). Environmental modifications

Environmental modifications will be applied to two departments (six wings). The environmental modifications will focus on facilitating daily physical activity and relaxation in the work environment, by changing the coffee corners, the open office environment, the meeting rooms and the entrance hall. Coffee corners will be adapted by adding bar chairs, magazines on the tables, an electronic dartboard, indoor plants, relaxing lights and by painting the coffee corner walls. In addition, the open office environment will be changed by introducing exercise balls, standing desks, sound masking to reduce background noise, and indoor plants. The interior of the meeting rooms will be changed by placing a stand-up table, a lamp, an indoor plant and by painting the walls. Furthermore, a ping-pong table and an indoor plant will be placed in the entrance hall. Prompts of decisions posters to promote daily physical activity and relaxation will be attached on the walls of the coffee corner and the entrance hall. In addition, food steps will be placed on the floor by the entrance hall to promote stair walking. All these environmental modifications will be promoted by posters in the coffee corner and in the hallway. Furthermore, the employees and the teamleaders will receive information by email about the environmental modifications.

# 4.5 Withdrawal of individual subjects

#

# 4.5.1 Specific criteria for withdrawal

Employees are able to withdraw at any time without reason.

#

# 4.6 Replacement of individual subjects after withdrawal

None

# 4.7 Follow-up of subjects withdrawn from treatment

Statistical analyses will be performed, based on the intention-to-treat principle. Subjects will remain in the analyses according to the allocated intervention or control group, after randomisation. Per-protocol analyses will be performed on all participants in the control group, and the participants in the GMI group, who have attended at least 70% of the GMI-sessions.

#

# 4.8 Premature termination of the study

As this trial is non-medical, but stimulates healthy behaviour instead, the study is not likely to be terminated prematurely.

#

# 5. Safety reporting

# 5.1 Section 10 WMO event

This is not an investigational medicinal study

# 5.2 Adverse and serious adverse events

This is not an investigational medicinal study.

#

# 5.3 Follow-up of adverse events

No serious adverse events from the questionnaires and the VIP program are expected. For this reason, no follow-up of adverse events will be performed. The VIP program consists of GMI-sessions, supported by a social media platform, and environmental modifications at the worksite. If employees encounter problems (mentally or physically) at any moment during the VIP program, teamleaders and/or the principal researcher will refer them to the occupational physician or the company’s social worker, who is physically present on the premises of the organisation.

# 5.4 Data Safety Monitoring Board (DSMB)

Not applicable.

# 6. Statistical analysis

First of all, baseline characteristics will be analyzed on differences between the four intervention arms, by two-way analysis of variance for numerical data and chi-square and logistic regression for categorical data. Multilevel regression analyses will be performed regarding the effectiveness of the intervention on need for recovery, the secondary outcome measures physical activity/relaxation and other work and health-related variables after 6 months (short-term) and 12 months (long-term). The intervention effect will be estimated in each stratum and a pooled estimated effect is calculated across strata. Multilevel analysis takes into account the clustering of observations of employees within the same wing. All analyses will be performed crude, and adjusted for possible confounders, such as gender and age. Also, these variables will be checked for effect modification. Correction for baseline values will be applied. Analyses will be carried out to test differences in sick leave between the four study intervention arms. All statistical analyses will be performed according to the intention-to-treat principle. Per protocol analyses will be performed to investigate the effects of the intervention in participants who showed high compliance (>70%) to the GMI intervention. Where necessary, missing data will be imputed using multiple imputation techniques. For all analyses a two tailed significance level of p<0.05 is considered statistically significant. All analyses will be performed with SPSS 19.0 (SPSS Inc. Chicago, Illinois, USA).

# 6.1 Economic evaluation

The aim of the economic evaluation will be to determine the cost-effectiveness, from a societal and company perspective, of the three intervention arms in comparison with the control group. Furthermore, the cost-utility from the company perspective will be studied. The follow-up will be one year, similar to the trial. The analyses will be performed according to the intention-to-treat principle. Productivity losses, i.e. sick leave and productivity data, will be measured with the Dutch version of the WHO-HPQ and IWPQ, (see secondary outcome measures), and the company registrations. In addition, participants are asked to complete a retrospective questionnaire, measuring health care use and personal costs related to the intervention. All questionnaires will be administered on a three-monthly basis. First, societal costs and company costs will be estimated, and compared between the intervention group and control group. Also, a cost-benefit analysis (CBA) will be performed in which the incremental intervention costs are compared to the incremental costs for productivity losses. Confidence intervals (95%) around the difference in costs will be obtained by bias corrected and accelerated (Bca) bootstrapping. Subsequently, a cost-effectiveness analysis (CEA) will be performed from the company perspective. An incremental cost-effectiveness ratio will be calculated, by dividing the difference between the mean company costs of the four intervention arms by the difference in the mean effects on the primary outcomes of the four intervention arms. Bootstrapped incremental cost-effectiveness ratios will be graphically presented on cost-effectiveness plans. Cost-acceptability curves will be generated, showing the probability for cost-effectiveness of the intervention at different ceiling ratios. Similarly, the CEA from the societal perspective will compare the incremental societal costs with the incremental effects on the primary outcomes. Sensitivity analyses will be done to assess the robustness of the results.

#

# 6.2 Process evaluation

A process evaluation at six months (at the end of the intervention) will be performed, by including questions on the context (organizational factors), recruitment (procedures to recruit teamleaders and employees), fidelity (whether the intervention is delivered as intended), reach (attendance rate), dose received (the extent the team leader and employees make use of the VIP program components and materials) and satisfaction about the intervention (attitude toward the VIP program). Teamleaders and employees will receive process evaluation questions, which concern subjects depending on whether they received the GMI-sessions and/or the environmental modifications. The employees in the wings with no intervention will not receive a process evaluation. Furthermore, managers of the wings in the intervention groups will be approached to give their opinion about the VIP-program.

In addition, after the six months intervention period, teamleaders will receive a questionnaire on which they will give their opinion about the GMI-sessions. Additionally, the quality of GMI-counselling is rated by means of the behavioural change counselling index (BECCI) (84). For this reason, the teamleader will be asked to record one GMI-session with their employees. Teamleaders in the wings with environmental modifications will be asked, by means of a process form, how their employees make use of the facilities to promote daily physical activity and relaxation.

# 7. Ethical considerations

# 7.1 Regulation statement

The study will be conducted according to the principles of the Declaration of Helsinki.

#

# 7.2 Objection by minors or incapacitated subjects

No minors or incapacitated subjects will participate in the current study.

#

# 7.3 Benefits and risk assessment, group relatedness

The VIP program involves GMI, supported by a social media platform. Main aim of GMI is to improve daily physical activity and relaxation of office employees. GMI is a group-related program, because it is a joint effort of both the teamleader and the employees to conduct the GMI-sessions. For optimal results, it is expected that everyone in a team participates. A teamleader will receive a two-day training in GMI. The skills learned during this training can be applied to other areas like yearly assessments of employees. After the training, the teamleader will lead a GMI-group, consisting of their own employees. GMI will be applied for 3 sessions of 90 minutes each, over a period of 6 weeks, and will end with a follow-up meeting of 90 minutes, 2 months after the final session. In the environmental modifications wings, the newly designed interior will facilitate daily physical activity and relaxation at work.

Participating in the VIP program poses minimal burden on both the intervention and control groups, i.e. three questionnaires (30-40 minutes) will be completed, during the study period of one year. Furthermore, two short questionnaires (WHO-HPQ and IWPQ) that gather data for economic analyses will be completed (5-10 minutes). For objective data on daily physical activity, a subsample of 100 employees will be asked to wear an accelerometer for one week and complete a brief daily log during the three measurement moments.

# 7.4 Compensation for injury

In this VIP project, there are no risks involved for individual employees and teamleaders. The intervention consists of GMI to promote daily physical activity and relaxation, supported by a social media platform. Also, environmental modifications will be made, which will not imply any risk on the participants. In the researchers’ opinion, no insurance is needed and therefore the researchers ask for dispensation for the insurance.

#

# 7.5 Incentives

To stimulate completion of the questionnaires, all employees and teamleaders will be ‘rewarded’ by incentives. At baseline, T1 and T2 a voucher for an in-company chair massage session will be offered by every 10^th^ participant returning the questionnaire. All employees will be offered little VIP gadgets, such as a pen or a sticker. Compliance to the T2 questionnaire could furthermore be promoted by organising special clinics, master classes and trials of sports.

#

# 8. Administrative aspects and publication

# 8.1 Handling and storage of data and documents

Questionnaire data will be gathered online.

# 8.2 Data entrance

Data obtained from the online questionnaires will be automatically directed into a coded SPSS file.

#

# 8.3 Privacy concerns

The principal researcher will equip the online version of the questionnaires with a 5-number person specific code. This researcher will store the person specific codes in an Excel file. By using this method, the researchers will be able to ensure that the employees’ data will be treated under code.

#

# 8.4 Data storage

Person-specific codes will be stored in an Excel database, secured by a code, and will be accessible only to the principal researcher, the research assistant, and an administrative assistant. Furthermore, the file that links the personal information of the employees (i.e. work e-mail addresses, names and/or workplace information) and the person-specific codes will be stored by and will be only accessible to the principal researcher. Data obtained from the questionnaires will be stored in a SPSS database by using the person-specific codes. The SPSS database will be secured by a code and will only be accessible to the principal researcher, the research assistant and an administrative assistant. The data on sick leave from the company will be gathered on person-specific codes. This will be done only when employees give their consent for that. By using this method, the researchers will be able to ensure the employees that their data will be treated anonymously.

#

# 8.5 Amendments

All substantial amendments will be notified to the METC and to the competent authority. Non-substantial amendments will not be notified to the accredited METC, but will be recorded and filed.

# 8.6 Annual progress report

The principal researcher will submit a summary of the progress of the trial to the accredited METC, once a year. Information will be provided on the date of inclusion of the first subject, numbers of subjects included and numbers of subjects, which have completed the trial, serious adverse events/ serious adverse reactions, other problems, and amendments.

# 8.7 End of study report

The principal researcher will notify the accredited METC of the end of the four-year study within a period of 8 weeks. In case the study is ended prematurely, the principal researcher will notify the accredited METC, including the reasons for the premature termination. Within one year after the end of the study, the researcher will submit a final study report with the results of the study, including any publications/abstracts of the study to the accredited METC.

#

# 8.8 Public disclosure and publication policy

The results of the study will be reported in at least five scientific articles, published in international, peer-reviewed journals. Further, practical implications of the research results will be described. Also, results will be presented at conferences, symposia and other meetings with both scientists and field experts. In consultation with the organisation that initiated this study, articles will be submitted to journals for health and safety professionals, employers and employees.

#

# Appendix 1: Design of the VIP project

Randomisation

Pre-stratification

Environmental modifications

(6 wings)

N=202

No environmental modifications

(7 wings)

N=202

GMI

(3 wings)

N=101

Non GMI

(3 wings)

N=101

GMI

(4 wings)

N=101

Non GMI

(3 wings)

N=101

13 wings

N=404

# Appendix 2: Flow Diagram of the VIP project

Kick-off VIP project for directors/managers

Kick-off meeting for teamleaders (N=80) + handing out informed consent cards

Collecting informed consent cards from teamleaders by VIP researcher

Sending baseline questionnaires

Non respons baseline questionnaires: …

No show:…

No show:…

No informed consent teamleaders: …

No informed consent employees: …

Randomisation

No GMI

Environmental modifications

Questionnaire Follow-up 6 months

GMI

Procedure

Trial

Pre stratification

No environmental modifications

Questionnaire Follow-up 12 months

# Appendix 3: Timeline of the VIP project

- **Follow-up Questionnaire 2**
- **Actigraph (100x)**
- **Social media platform + environmental modifications can be removed**
- **Final GMI-session**
- **Follow-up Questionnaire 1**
- **Process evaluation**
- **Actigraph (100x)**
- **Short Questionnaire**
- **Short Questionnaire**
- **Baseline Questionnaire**
- **Actigraph (100x)**
- **Randomization**
- **Environmental modifications**
- **Start GMI-sessions**
- **Social media platform**

**References**

(1) Eurofound. Changes over time – **First findings from the fifth European Working Conditions Survey**: *Résumé*. 2010.

(2) Van Amelsvoort LG, Kant IJ, Bultmann U, Swaen GM: **Need for recovery after work and the subsequent risk of cardiovascular disease in a working population.** *Occup Environ Med* 2003, **60:** 83-87.

(3) Macfarlane GJ, Pallewatte N, Paudyal P, Blyth FM, Coggon D, Crombez G: E**valuation of work-related psychosocial factors and regional musculoskeletal pain: results from a EULAR Task Force.** *Ann Rheum Dis* 2009, **68:** 885-91.

(4) Choi B, Ostergren PO, Canivet C, Moghadassi M, Lindeberg S, Karasek R: **Synergistic interaction effect between job control and social support at work on general psychological distress.** *Int Arch Occup Environ Health* 2011, 84:77-89.

(5) Sluiter JK, de Croon EM, Meijman TF, Frings-Dresen MH: N**eed for recovery from work related fatigue and its role in the development and prediction of subjective health complaints.** *Occup Environ Med* 2003, **60:** 62-70.

(6) Meijman T.F., Mulder G: P**sychological aspects of workload.** In: Drenth PJD, Thierry H, editors. Handbook of work and organizational psychology. Psychology Press; 1998. 5-33.

(7) Van Veldhoven M: Need for recovery after work. A**n overview of construct, measurement and research.** In: Houdmont J, Leka S, editors. Occupational health psychology. European perspectives on research, education and practice. Nottingham: Nottingham University Press; 2008.

(8) Stewart J, Janicki D, Kamarck T: **Cardiovascular reactivity to and recovery from psychological challenge as predictors of 3-year change in blood pressure.** *Health Psychol* 2006, **25:**111-118.

(9) Sluiter JK, Van der Beek AJ, Frings-Dresen MHW. **The influence of work characteristics on the need for recovery and experienced health: A study on coach drivers.** *Ergonomics* 1999, **42:** 573-83.

(10) Geurts SAE, Sonnentag S: **Recovery as an explanatory mechanism in the relation between acutestress reactions and chronic health impairment.** *Journal of Work and Environmental Health* 2006,**32:**482-92.

(11) Meijman TF, Mulder G: **Psychological aspects of workload.** In: Drenth PJD, Thierry H, editors. Handbook of work and organizational psychology.Hove: Psychology Press; 1998.

(12) Etzion D, Eden D, Lapidot Y: **Relief from job stressors and burnout: Reserve service as a respite.** *Journal of Applied Psychology* 1998, **83:**577-85.

(13) Grebner S, Semmer NK, Elfering A: **Working conditions and three types of well-being: a longitudinal study with self-report and rating data.** *Journal of Occupational and Organizational Psychology* 2010, **10:**31-43.

(14) Sonnentag S, Bayer U: **Switching off mentally: predictors and consequences of psychological detachment from work during off-job time.** *Journal of Occupational Health Psychology* 2005,**10:**393-414.

(15) Muraven M, Baumeister RF: **Self-regulation and depletion of limited
resources: Does self-control resemble a muscle?** *Psychological Bulletin* 2000, **126:** 247-259.

(16) Trougakos JP, Beal DJ, Green SG, Weiss HM: **Making the break count: An episodic examination of recovery activities, emotional experiences, and affective
delivery.** *Academy of Management Journal* 2008, **51:**131-1146.

(17) Sonnentag S: **Work, recovery activities, and individual well-being: A diary study.** *Journal of Occupational Health Psychology* 2001, **6:** 196-210.

(18) Sonnentag S, Kuttler I, Fritz C: **Job stressors, emotional exhaustion, and need for recovery: A multi-source study on the benefits of psychological detachment.** *Journal of Vocational Behavior* 2010,**76:**355-65.

(19) Sonnentag S, Binnewies C, Mozja EJ: **Staying Well and Engaged when Demands are High: The Role of Psychological Detachment.** *Journal of Applied Psychology* 2010, 95: 965-976.

(20) Bellarose C, Chen PY: **The effectiveness and practicality of occupational stress management interventions: A survey of subject matter expert opinions.** J*ournal of Occupational Health Psychology* 1997, **2:** 247-62.

(21) Norris R, Carroll D, Cochrane R: **The effects of aerobic and anaerobic training
on fitness, blood pressure, and psychological stress and well-being.** *J Psychosom Res* 1990, **34:**367-75.

(22) Lechner L, de Vries H: **Effects of an Employee Fitness Program on Reduced Absenteeism.** *Journal of Occupational and Environmental Medicine* 1997, **39:** 827-31.

(23) Van den Heuvel SG, Boshuizen HC, Hildebrandt BM, Blatter GA, Arriens GA, Bongers PM: **Effect of sporting activity on absenteeism in a working population.** *Br J Sports Med* 2005,**39:**1-5.

(24) Rosenfeld O, Tenenbaum G, Ruskin H: **Behavioural modifications following a physical activity programme in the Israeli pharmaceutical industry.** *Aust J Sci Med Sport* 1990, **22:** 93-6.

(25) Shephard RJ: **Exercise and relaxation in health promotion.** *Sports Med* 1997, **23:** 211-7.

(26) Benson H. **The relaxation response.** New York: Morrow; 1975.

(27) Stone AA, Kennedy-Moore E, Neale JM: A**ssociation between daily coping and end-of-day mood.** *Health Psychology* 1995,**14:**341-9.

(28) Burns L. **Relaxation in the management of stress.** In: Marshall J, Cooper C, editors. In coping with stress.London: Gower; 1981.

(29) Bartholomew LK, Parcel GS, Kok G, Gottlieb NH. Pl**annning health promotion programs: an intervention mapping approach.** San Francisco, CA: Jossey- Bass; 2006.

(30) Abramowitz S, Flattery D, Franses K, Berry L: **Linking a Motivational Interviewing Curriculum to the Chronic Care Model.** *J Gen Intern Med* 2010, **25:** 620-626. 2

(31) Butterworth S, Linden A, McClay W, Leo M: **Effect of Motivational Interviewing-Based Health Coaching on Employees' Physical and Mental Health Status.** J*ournal of Occupational Health Psychology* 2006, **11:** 358-365.

(32) Elliot D, Goldberg L, Kuehl K, Moe E, Bregner R, Pickering M: **The PHLAME (Promoting Healthy Lifestyles: Alternative Models' Effects) Firefighter Study: Outcomes of Two Models of Behavior Change.** *J Occup Environ Med* 2007, **49:** 204-213.

(33) Groeneveld I, Proper K, van der Beek A, van Mechelen W: **Sustained body weight reduction by an individual-based lifestyle intervention for workers in the construction industry at risk for cardiovascular disease: results of a randomized controlled trial.** *Prev Med* 2010, **51:** 240-246.

(34) Schneider R, Casey J, Kohn R: M**otivational versus confrontational interviewing: a comparison of substance abuse assessment practices at employee assistance programs.** *J Behav Health Serv Res* 2000, **27:** 60-74.

(35) Bailey K, Webster R, Lewin T: **Pilot Randomized Controlled Trial of a Brief Alcohol Intervention Group for Adolescents.** *Drug and Alcohol Review* 2004, **23:** 157-166.

(36) Foote J, DeLuca A, Magura S, Warner A, Grand A, Rosenblum A, et al: A **Group Motivational Treatment for Chemical Dependency.** *Journal of Substance Abuse Treatment* 2010, **17:**181-92.

(37) Ingersoll K, Wagner C, Gharib S: M**otivational Groups for Community Substance Abuse Programs.** Mid-Atlantic Addiction Technology Transfer Center
Unifying Science, Education and Services to Transform Lives ; 2000.

(38) Lincourt P, Kuettel T, Bombardier C: **Motivational interviewing in a group setting with mandated clients: A pilot study.** *Addictive Behaviors* 2002, **27:**381-91.

(39) Van Horn D, Bux D: **A pilot test of motivational interviewing groups for dually
diagnosed inpatients.** *Journal of Substance Abuse Treatment* 2001, **20:**191-5.

(40) Velasquez M, Stephens N, Ingersoll K: **Motivational interviewing in groups.** *Journal of Groups in Addiction & Recovery* 2006, **1:** 27-50.

(41) Deci E, Ryan R. **Handbook of self determination research.** Rocheste, NY: University of Rochester Press; 2002.

(42) Miller WR, Rollnick S. **Motivational Interviewing: Preparing people for change.** New York: Guilford Press; 2002.

(43) Abood D, Black D, Feral D. **Nutrition Education Worksite Intervention for University Staff:Application of the Health Belief Model**. J Nutr Educ Behav 2003;35:260-7.

(44) Rush E, Cumin M, Migriauli L, Ferugson L, Planks L. **One Year Sustainability of Risk Factor Change from a 9-WeekWorkplace Intervention**. Journal of Environmental and Public Health 2009;2:1-7.

(45) Paroutis S, Al Saleh A: D**eterminants of knowledge sharing using Web 2.0 technologies.** *Journal of Knowledge Management* 2009,**13:**52-63.

(46) Engbers L, van Poppel M, China Paw M, van Mechelen W. **Worksite Health Promotion Programs with Environmental Changes.** *Am J Prev Med* 2005, **29:**61-70.

(47) Weiss H, Cropanzano R: **An effective events approach to job satisfaction. In: Staw B, Cummings L, editors.** Research in organizational behavior. Greenwich: CT: JAI Press; 1996.

(48) Veitch J, Newsham G, Boyce P, Jones C: **Lighting appraisal, well-being, and performance in open-plan offices: a linked mechanisms approach.** *Lighting Research and Technology* 2008, **40:**133-51.

(49) Croon E, Sluiter J, Kuijer P, Frings-Dresen M: T**he effect of office
concepts on worker health and performance: a systematic review of the literature.** *Ergonomics* 2005, **48:**119-34.

(50) Haynes B: **The impact of the behavioural environment on office productivity. J***ournal of Facilities Management* 2007, **5:**158-171.

(51) Olsen J: **Research about office workplace activities important to US business-and how to support them.** *Journal of Facilities Management* 2002,**1:**31-47.

(52) Vink P, de Korte E, Blok M, Groenesteijn L: **Effects of the Office Environment on Health and Productivity 1:Effects of Coffee Corner Position.** *Ergonomics and Health Aspects* 2007, **17:** 157-62.

(53) Cohen M, Rogelberg S, Allen J, Luong A: **Meeting Design Characteristics and Attendee Perceptions of Staff/Team Meeting Quality.** *Group Dynamics: Theory, Research, and Practice* 2011, **1:** 90-104.

(54) Leach D, Rogelberg S, Warr P, Burnfield J: P**erceived meeting effectiveness: The role of design characteristics.** *Journal of Business and Psychology* 2011, **24:** 65-76.

(55) Bluedorm A, Turban D, Love M: **The effects of stand-up and sit-down meeting formats on meeting outcomes.** J*ournal of Applied Psychology* 1999, **84:** 277-285.

(56) Kwallek N, Lewis C, Robbins A. **Effects of office interior color on workers' mood and productivity.** *Perceptual and Motor Skills* 1988, **66:**123-8.

(57) Kwallek N, Lewis C, Lin-Hsiao J, Woodson H. **Effects of nine monochromatic office interior colors on office clerical tasks and worker mood.** C*olor Research and Application* 1996, **21:**448-58.

(58) Evans G, Johnson D. **Stress and Open-Office Noise.** *Journal of Applied Psychology* 2000, **85:**779-83.

(59) Banburry S, Berry D: **Office noise and employee concentration: identifying causes of disruption and potential improvements.** *Ergonomics* 2005, **48:**25-37.

(60) Nachtegaal J, Kuik D, Anema J, Goverts S, Festen J, Kramer S: **Hearing status, need for recovery after work, and psychosocial work characteristics: results from an Internet based National Survey on Hearing.** *International Journal of Audiology* 2009, **48:**684-91.

(61) Bakker I, Van der Voordt T: **The influence of plants on productivity: A critical assessment of research findings and test methods.** *Facilities* 2010; **28:**416.

(62) Shibata S, Suzuki N: **Effects of an indoor plant on creative task performance and mood.** *Journal of Environmental Psychology* 2002, **27:** 265-72.

(63) Van Veldhoven M, Broersen S: M**easurement quality and validity of the "need for recovery scale.** *Occupational and Environmental Medicine* 2003, **60:**3-9.

(64) De Croon E, Sluiter J, Frings-Dresen M: **Psychometric properties of the Need for Recovery after work scale: test-retest reliability and sensitivity to detect change.** *Occupational and Environmental Medicine* 2006, **63:**202-206.

(65) Kuijer P, Van der Beek AVDJ, Visser B, Frings-Dresen M: **Effect of job rotation on need for recovery, musculoskeletal complaints, and sick leave due to musculoskeletal complaints: a prospective study among refuse collectors.** *American Journal of Industrial Medicine* 2005, **47:**394-402.

(66) Kelder S, Jacobs D, Jeffery R, McGovern P, Forster J: Th**e worksite component of variance: design effects and the Healthy Worker Project.** *Health Educ Res* 1993, **8:**555-566.

(67) Martinson B, Murray D, Jeffery R, Henrikus D: **Intraclass correlation for measures from aworksite health promotion study: Estimates, correlates and applications.** *American Journal of Health Promotion* 1999,**13:**347-357.

(68) Murray D, Blitstein J: M**ethods to reduce the impact of intraclass correltaion in group-randomized trials.** *Evaluation Review* 2003, **27:**79-103.

(69) Wendel-Vos GCW, Schuita AJ, Sarisc WHM, Kronmhout D: **Reproducibility and relative validity of the Short Questionnaire to Assess Health-enhancing physical activity.** *Journal of Clinical Epidemiology* 2003, **56:**1163-1169.

(70) Van Poppel M, Chinapaw M, Mokkink L, van Mechelen W, Terwee C: **Physical Activity Questionnaires for AdultsA Systematic Review of Measurement Properties.** *Sports Med* 2011, **40:** 565-600.

(71) Sonnentag S, Fritz C: **The Recovery Experience Questionnaire: Development and Validation of a Measure for Assessing Recuperation and Unwinding From Work.** *Journal of Occupational Health Psychology* 2007, **12:** 204-221.

(72) De Bloom J, Geurts SAE, Kompier MAJ: T**he effect of vacation activities and experiences on employee health and well being.** manuscript submitted for publication 2010.

(73) Demerouti E, Bakker A, Vardakou I, Kantas A: **The convergent validity of two burnout instruments: A multitrait-multimethod analysis.** *European Journal of Psychological Assessment* 2003,**19:**12-23.

(74) Van der Zee K, Sanderman R: Het meten van gezondheidstoestand met de RAND-36: een handleiding. Groningen: *Noordelijk Centrum voor Gezondheidsvraagstukken* : 1993.

(75) Cohen S, Kamarchk T, Mermelstein: **A global measure of perceived stress.** J*ournal of Health and Social Behavior* 1983, **24:**385-96.

(76) Lakerveld J, Bot S, Chinapaw M, Knol D, de Vet H, Nijpels G: Me**asuring pathways towards a healthier lifestyle in the Hoorn Prevention Study: the determinants of lifestyle behavior questionnaire (DLBQ).** unpublished manuscript 2010.

(77) De Vries H, Kremers SP, Smeets T, Brug J, Eijmael K: **The effectiveness of tailored feedback and action plans in an intervention addressing multiple health behaviours.** *American Journal of Health Promotion* 2008, **22:** 417-425.

(78) McEachan RC, Lawton RJ, Jackson C, Conner M, Lunt J: **Evidence, Theory and Context: Using intervention mapping to develop a worksite physical activity intervention.** B*MC Public Health* 2008, **8:** 326-338.

(79) Kessler R, Barber C, Beck A, Berglund P, Cleary P, McKenas D. **The World Health Organization Health and Work Performance Questionnaire (HPQ).** *J Occup Environ Med* 2003, **45:**156-74.

(80) Koopmans L, Bernaards C, Hildebrandt V, Schaufeli W, de Vet H, Van der Beek A: **Conceptual frameworks of individual work performance - A systematic review.** Submitted to *Journal of Occupational and Environmental Medicine* 2011.

(81) Karasek R, Brisson C, Kawakami N, Houtman I, Bongers P, Amick B: T**he Job content Questionnaire (JCQ): an instrument for internationally comparative assessments of psychosocial job characteristics.** *J Occup Health Psychol* 1998, **3:**322-55.

(82) Karasek R, Theorell T. Healthy Work: **Stress , Productivity , and the Reconstruction of Working Life.** New York : Basic Books: 1990.

(83) Prochaska JO, DiClemente CC: **Stages and processes of self-change of smoking: toward an integrative model of change.** *Journal of Consulting and Clinical Psychology* 1983, **51:**390-395.

(84) Lane C, Huws-Thomas M, Hood K, Rollnick S, Edwards K, Robling M: M**easuring adaptations of motivational interviewing: the development and validation of the behavior change counseling index (BECCI).** *Patient Education and Counselling* 2005, **56:**166-73.
